# Identifying time‐resolved features of nocturnal sleep characteristics of narcolepsy using machine learning

**DOI:** 10.1111/jsr.14216

**Published:** 2024-04-26

**Authors:** Marco Vilela, Brian Tracey, Dmitri Volfson, Lucie Barateau, Alice Cai, Derek L. Buhl, Yves Dauvilliers

**Affiliations:** ^1^ Takeda Development Center Americas, Inc. Cambridge Massachusetts USA; ^2^ Department of Neurology, Sleep‐Wake Disorders Center, Gui‐de‐Chauliac Hospital, CHU Montpellier France; ^3^ National Reference Network for Narcolepsy Montpellier France; ^4^ Institute for Neurosciences of Montpellier (INM), INSERM University of Montpellier Montpellier France

**Keywords:** disrupted nighttime sleep, idiopathic hypersomnia, machine learning, narcolepsy, nocturnal polysomnography, sleep architecture

## Abstract

The differential diagnosis of narcolepsy type 1, a rare, chronic, central disorder of hypersomnolence, is challenging due to overlapping symptoms with other hypersomnolence disorders. While recent years have seen significant growth in our understanding of nocturnal polysomnography narcolepsy type 1 features, there remains a need for improving methods to differentiate narcolepsy type 1 nighttime sleep features from those of individuals without narcolepsy type 1. We aimed to develop a machine learning framework for identifying sleep features to discriminate narcolepsy type 1 from clinical controls, narcolepsy type 2 and idiopathic hypersomnia. The population included polysomnography data from 350 drug‐free individuals (114 narcolepsy type 1, 90 narcolepsy type 2, 105 idiopathic hypersomnia, and 41 clinical controls) collected at the National Reference Centers for Narcolepsy in Montpelier, France. Several sets of nocturnal sleep features were explored, as well as the value of time‐resolving sleep architecture by analysing sleep per quarter‐night. Several patterns of nighttime sleep evolution emerged that differed between narcolepsy type 1, clinical controls, narcolepsy type 2 and idiopathic hypersomnia, with increased nighttime instability observed in patients with narcolepsy type 1. Using machine learning models, we identified rapid eye movement sleep onset as the best single polysomnography feature to distinguish narcolepsy type 1 from controls, narcolepsy type 2 and idiopathic hypersomnia. By combining multiple feature sets capturing different aspects of sleep across quarter‐night periods, we were able to further improve between‐group discrimination and could identify the most discriminative sleep features. Our results highlight salient polysomnography features and the relevance of assessing their time‐dependent changes during sleep that could aid diagnosis and measure the impact of novel therapeutics in future clinical trials.

## Graphical abstract

Changes in sleep architecture on nocturnal polysomnography based on whole‐night and quarter‐night sleep metrics could distinguish narcolepsy type 1 (NT1) from clinical controls, narcolepsy type 2 (NT2) and idiopathic hypersomnia (IH). Optimal discriminating performance was achieved with a combination of different features across quarter‐night periods, with early rapid eye movement (REM) features ranked as highly important.

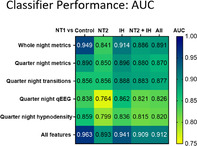

## 1 INTRODUCTION

Narcolepsy type 1 (NT1), a rare, chronic, central disorder of hypersomnolence (CDH), is characterized by excessive daytime sleepiness (EDS), cataplexy, disrupted nighttime sleep (DNS), hypnagogic/hypnopompic hallucinations and sleep paralysis (Roth et al., 2013). Diagnosis is typically based on clinical history, polysomnography (PSG) and multiple sleep latency test (MSLT) results. However, some hallmarks of NT1 are shared with other CDH, such as narcolepsy type 2 (NT2; also known as narcolepsy without cataplexy) and idiopathic hypersomnia (IH; characterized by the presence of EDS, and sometimes sleep inertia and long nighttime sleep, with unknown origin), making the differential diagnosis challenging.

Several differentiating characteristics for CDHs have been identified. For example, the HLA‐DQB1*06:02 allele is strongly associated with NT1 (Capittini et al., 2018). Low cerebrospinal fluid (CSF) orexin (hypocretin) concentration (< 110 pg/ml) is a highly specific and sensitive biomarker for NT1 (Mignot et al., 1992; Taheri et al., 2002), while patients diagnosed with NT2 have, by definition, CSF orexin concentrations above 110 pg/ml. However, few physicians report incorporating genetic testing or CSF orexin concentration measurement into routine assessments of the hypersomnolence condition (Rosenthal et al., 2021), and no reliable biomarkers for NT2 or IH—genetic or otherwise—have been discovered to date. IH, like NT2, has normal CSF orexin concentrations, and differs from NT1 and NT2 primarily due to the absence of two or more sleep‐onset rapid eye movement (REM) periods (SOREMPs; i.e. REM periods occurring within 15 min of sleep onset) on PSG and MSLT. However, poor reproducibility of MSLT results was demonstrated in participants diagnosed with NT2 and IH (Ruoff et al., 2018; Trotti et al., 2013).

Differences in nighttime sleep architecture have been reported between NT1, NT2 and IH; most notably, orexin deficiency in NT1 may lead to changes in the evolution of sleep architecture and continuity throughout the night that could separate patients with NT1 from individuals with intact orexin systems, for example, patients with NT2 or IH (Barateau et al., 2020). Focus has been directed towards identifying nocturnal PSG findings characteristic of NT1. For instance, the presence of SOREMPs, increased sleep instability reflected by an increased amount of N1 sleep, longer wake after sleep onset (WASO), decreased sleep efficiency, and increased numbers of wake bouts, sleep bouts and sleep stage transitions are all PSG features of NT1 (Barateau et al., 2020). Moreover, typical discrete states of sleep are shifted to “mixed states”, reinforcing the hypothesis that the mechanisms underlying sleep stability are affected in NT1 (Hansen et al., 2017; Maski et al., 2021; Maski et al., 2022; Saper et al., 2005). DNS is a cardinal feature of NT1 (Barateau et al., 2022; Maski et al., 2022), although not required for diagnosis based on International Classification of Sleep Disorders (ICSD‐3) criteria (American Academy of Sleep Medicine, 2014), and affects approximately 50% of individuals with NT2, whereas individuals with IH have normal sleep and sometimes exhibit a prolonged total sleep time (TST; Blattner & Maski, 2023; Dauvilliers et al., 2022). Nocturnal PSG testing, used for the routine assessment of sleep disorders, could therefore help differentiate NT1, NT2 and IH.

The application of machine learning techniques to construct predictive models for the diagnosis of sleep disorders has been investigated in several recent studies, including a machine learning‐based predictive model for NT1 versus NT2 using European Narcolepsy Network (EU‐NN) data (Zhang et al., 2018) and automated sleep scoring of PSG data in 30‐s sleep epochs to reliably classify sleep stages (Sharma et al., 2021). Recent work showed good performance in the automated detection of NT1 using PSG data (Cesari et al., 2022; Stephansen et al., 2018), which may enable future diagnostic applications. However, these recent papers exploit relatively complex features with limited interpretability, potentially limiting their clinical application.

Nocturnal sleep continuity and architecture, including REM sleep, varies throughout the night and can be best observed using time‐resolved sleep analysis in combination with machine learning. This strategy could provide unique, clinically relevant features that increase discrimination between NT1, NT2, IH and clinical controls. To test this approach, we built machine learning models using sleep metrics extracted in a time‐dependent fashion, by quarter‐night periods to account for changes in sleep parameters throughout the night, to provide additional dynamic information for the classifier. We further analysed multiple feature sets capturing different aspects of sleep, focusing on interpretable features (e.g. time in sleep state metrics, sleep state transition, qEEG [quantitative electroencephalogram]), and used classification performance to reveal aspects of sleep that are most discriminative for NT1 versus clinical controls and other CDH. Our main aims were to identify clinically interpretable sleep features that characterize NT1 from other CDHs, and to further establish methods for CDH classification.

## 2 METHODS

### 2.1 Population

The study included data from 350 adults with confirmed NT1 (*n* = 114; 69 male; mean age 37.0 ± 14.5 years), NT2 (*n* = 90; 51 male; mean age 30.0 ± 10.8 years) or IH (*n* = 105; 25 male; 30.3 ± 10.6 years), and 41 non‐CDH clinical controls with complaints of daytime sleepiness but without objective hypersomnolence disorder (20 male; mean age 34.4 ± 12.0 years), all of whom were referred to the Sleep Disorder Unit and the National Reference Center for Rare Hypersomnias, Montpellier, France between 2006 and 2020. Participants were diagnosed according to ICSD‐3 criteria (American Academy of Sleep Medicine, 2014). All participants underwent the same standardized screening evaluation, including medical interview by a sleep expert, and video‐PSG followed by MSLT. All patients with IH additionally underwent a 32‐hr bed‐rest PSG recording, as previously described (Evangelista et al., 2018), during a separate visit, which confirmed a TST > 19/32 hr (long sleep phenotype) for 87 (82.9%) patients. However, all data analysed for the present study come from the first nighttime PSG with a recording time of 23:00 hours to 07:00 hours (i.e. 8 hr maximum recording). Clinical controls were required to have an MSLT mean sleep latency > 8 min, with no evidence of cataplexy, excessive quantity of sleep, narcolepsy or IH based on ICSD‐3 criteria, sleep efficiency ≥ 70%, apnea–hypopnea index (AHI) < 15 per hr, periodic leg movements index (PLMI) < 15 events per hr, and no other sleep disorders (see Table S1 for AHI and PLMI statistics by group). All participants were drug‐naïve or had discontinued drugs targeting the central nervous system with effects on sleep (e.g. withdrawal of stimulants, anti‐cataplectics) 3–5 weeks prior to evaluation, and remained untreated throughout the study period. CSF orexin‐A concentrations were measured in 209 participants in duplicate from each sample using the I^125^‐radioimmunoassay (RIA) kit from Phoenix Pharmaceuticals, following the manufacturer's recommendation. All values were back‐referenced to the Stanford reference samples. Low CSF orexin‐A concentrations (< 110 pg/ml) were reported in all patients with NT1 (*n* = 100), with intermediate concentrations (110–200 pg/ml) in 10 patients with NT2, and normal concentrations (> 200 pg/ml) in 51 patients with NT2, 41 with IH and seven clinical controls.

The study was approved by the institutional ethics committees (Comité de Protection des Personnes, France; “Constitution of a cohort and of a clinical, neurophysiological and biological bank of rare hypersomnolence disorders” SOMNOBANK). All participants provided written informed consent.

### 2.2 Nocturnal PSG assessment

All participants underwent a standard PSG in the sleep laboratory. PSGs used AASM standard leads, including EEG leads (central C3, C4; occipital O2, each referenced to contralateral mastoid), left and right electrooculogram, chin electromyogram (EMG; three mandible leads, two used as reference derivation, with the third as backup in the event of data quality issues), cannula/pressure transducer system, mouth thermistor, chest and abdominal bands, pulse oximeter, EMG electrodes on bilateral anterior tibialis muscles, and electrocardiogram. Sleep scoring was performed by expert sleep physicians according to standard criteria of the AASM, blinded to clinical features and before final diagnosis. Recorded PSG variables were TST, sleep efficiency, percentage of time spent in sleep stages (N1, N2, N3 and REM), sleep latency, REM sleep latency, WASO, AHI, periodic leg movements during sleep and the micro‐arousal index. Sleep apneas were defined as a drop in peak flow signal excursion by ≥ 90% for ≥ 10 s, and hypopneas as a drop in the peak flow signal excursion by ≥ 30% for ≥ 10 s associated with either a ≥ 3% desaturation and/or a micro‐arousal (Iber, Ancoli‐Israel, Chesson, Quan, & Medicine, 2007).

### 2.3 Extraction of PSG features

Five PSG feature sets were computed from PSG data (Table 1). Automatically scored hypnograms were initially considered; however, they were later discarded due to false‐positive REM scoring at the beginning of the night. This type of error can cause classification problems as early‐night REM is a strong indicator of NT1. To remove uncertainties related to automated sleep scoring, manually scored hypnograms were used for analysis; sleep scoring from anonymized PSG data was blinded to CDH analysis. For all feature sets, nocturnal PSG was restricted from persistent sleep onset defined for this study as at least 5 min of continuous sleep (Morin et al., 2009; Smits et al., 2003) to final end‐of‐sleep epoch. For the first feature set, *whole‐night sleep metrics* including REM sleep onset and fraction of time in each sleep stage were computed from manually scored hypnograms. Stage shift index (SSI), defined as number of changes between sleep stage per hour between persistent sleep onset and final awakening, was included as a measure of sleep fragmentation (Laffan et al., 2010). The other four feature sets shown in Table 1 were computed for four equal quarter‐night periods (equally dividing the time from persistent sleep onset to final awakening) to test whether time‐resolved, dynamic information about the features is relevant in discriminating NT1 from clinical controls, NT2 and IH. The value of sleep resolution by quarter‐night periods was recently reported (Cesari et al., 2022; Vilela et al., 2022). *Quarter‐night sleep metrics* captured standard sleep metrics in a time‐resolved fashion, while *quarter‐night transition matrices* captured transitions between sleep states.

**TABLE 1:** List of feature sets and corresponding features

Feature set	Time resolution	Features	Number of features
Sleep Metrics	Whole‐night	Proportion in each of the 4 sleep stages (N1, N2, N3, REM) and W; TST; SSI; REM sleep onset	8
Sleep Metrics	Quarter‐night	Proportion in each of the 4 sleep stages and W; TST; SSI	28 (7 per quarter‐night)
Transition Matrix	Quarter‐night	Transition matrix from any sleep stage to any stage	100 (25 per quarter‐night)
qEEG	Quarter‐night	Power bands: delta (0.5–4 Hz); theta (4–8 Hz); alpha (8–12 Hz); sigma (12–16 Hz); beta (16–30 Hz); gamma (30–47 Hz)	120 (6 per quarter‐night for each of the 5 sleep stages)
Hypnodensity	Quarter‐night	Probabilities for all sleep stages and their entropy + the joint probabilities of all combinations of any two stages	64 (16 per quarter‐night)
All features	Quarter‐ and whole‐night	All features of each feature set	320

qEEG, quantitative electroencephalogram; REM, rapid eye movement; SSI, stage shift index; TST, total sleep time; W, wake.

*Quarter‐night qEEG features* were computed, consisting of the power bands described in Table 1, conditional on sleep state. qEEG band powers were calculated from the C3 channel using multi‐taper power spectral density analysis (Babadi & Brown, 2014). Mean relative power across non‐overlapping 2‐s slices over each 30‐s epoch was calculated for each band by dividing the power within that band by the total power summed across all bands of interest (0.5–47 Hz). Slices that contained any clipping (above or below maximum recording capabilities) or flat‐line data (three or more consecutive samples with the same value) were rejected, and spectra were calculated using the remaining slices. Epochs manually marked as “no‐score” due to arousals or high noise levels were excluded from the analysis.

*Quarter‐night hypnodensity metrics* were computed using a deep learning model (Stephansen et al., 2018) trained to mimic the sleep stage scoring provided by human experts following the rules of the AASM Scoring Manual (Iber et al., 2007). The model outputs probabilities of all sleep stages within each epoch, with the collection of these probabilities referred to as a hypnodensity. For consistency with other features, we averaged the hypnodensities from their native 15‐s epoch length down to 30 s epochs. We used the marginal probabilities for all five states as features (estimated probability of being in each state) and all possible mixtures of any two states (computed as products of pairwise probabilities). To include an overall measure of mixed states, we also computed the entropy of the stage probabilities. These hypnodensity features were chosen for their interpretability and are a subset of those examined in Stephansen et al. (2018).

### 2.4 Classifier framework

**FIGURE 1:**
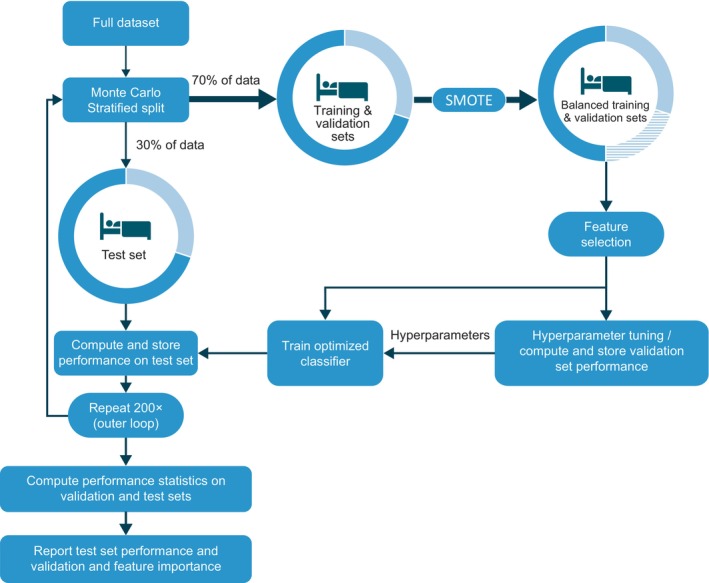
Schematic overview of the classification process. Data were divided into train/test and validation sets. The train/test set was augmented to balance the classes examples then used for feature selection and classifier optimization. Final performance metrics were computed with the validation set. AUC, area under the receiver operating characteristic curve; NT1, narcolepsy type 1; SD, standard deviation; SMOTE, synthetic minority over‐sampling technique.

The machine learning framework consists of an outer resampling loop and inner training/testing loop (Figure 1). Five classification tasks were established: differentiation between NT1 versus clinical controls; NT1 versus NT2; NT1 versus IH; NT1 versus NT2 + IH; and NT1 versus All (NT2 + IH + clinical controls). For each task, three classification models were tested: XGBoost (Friedman, 2002); Random Forest (RF; Breiman, 2001); and a Gaussian Process (GP; Murphy, 2012). The model training and evaluation process was repeated separately for each classification task and classification model. Detailed methods are available in the Supplementary Methods.

### 2.5 Statistical analysis

In addition to the machine learning framework described above, two statistical analyses were performed on extracted sleep features, as an independent check on how individual endpoints differ across diagnostic groups. Importantly, there was no linkage between these statistical analyses and the machine learning modelling. First, differences between groups for all extracted features were evaluated using an ANOVA test controlling for age, with false discovery rate adjustments to *p*‐values. Tukey's multiple comparison was consequently applied to expose pairwise differences among groups. Second, for quarter‐night features only, a linear mixed model was used to explore whether the time evolution of individual sleep features varies by diagnosis (see Supplementary Methods). For both analyses, significance was set at *p* < 0.05 and analyses were performed using R version 4.2.0.

## 3 RESULTS

### 3.1 Evaluation of PSG features

**FIGURE 2:**
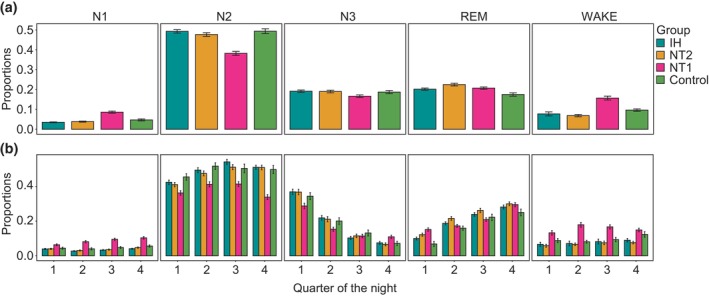
Between‐group (NT1, NT2, IH and clinical controls) differences in polysomnographic (PSG) quantification of sleep stages observed in both whole‐night and quarter‐night periods. The proportion of time spent in each sleep stage computed for: (a) whole‐night period; and (b) each quarter of the night. Bar plots show the mean and standard deviation. ANOVA results are listed in Table S1 (whole‐night sleep metrics) and Table S2 (quarter‐night sleep metrics). IH, idiopathic hypersomnia; NT1, narcolepsy type 1; NT2, narcolepsy type 2.

Differences in the proportion of time in each sleep stage were observed to depend on diagnosis on both whole‐night (Figure 2a) and quarter‐night evaluations (Figure 2b). PSG data from individuals with NT1 showed a significantly greater proportion of time in N1 and wake stages, and a lower proportion of time in N2 stage on whole‐night sleep metrics compared with NT2, IH and controls (Figure 2a; Table S2). Across quarter‐night periods, the proportion of time in N1 increased from Q1 to Q4 for individuals with NT1 while remaining constant for IH, NT2 and controls, while time in REM sleep was significantly greater during the first quarter with NT1 than with IH and controls (Figure 2b; Table S3).

Mixed model results showed that many PSG features evolve differently across quarters of the night depending on group (NT1, NT2, IH and clinical controls). Features exhibiting these group‐dependent temporal patterns include the fraction of time spent in each sleep state, as well as many sleep state transition, hypnodensity and qEEG features. A full list of features with significant quarter‐night by diagnosis interactions is given in the Supplementary Materials and summarized in Table S7.

### 3.2 Evaluation of classification models

We tested all feature sets for their discriminatory power and overall importance as input for the five classifications (i.e. NT1 versus clinical controls; NT1 versus NT2; NT1 versus IH; NT1 versus NT2 + IH; and NT1 versus All). Based on the mean F1 value in the test set over 200 independent runs, the RF classifier was chosen as it was the best performer across all classification tasks when all features were combined (Figure S9 for NT1 versus clinical controls), and thus all findings reported were obtained using this method. Training/validation and test performances are compared in Table S8. Performance statistics including F1 score, area under the precision‐recall curve (AUC_PR) and area under the receiver operating curve (AUC_ROC) are listed in Table 2 for all feature sets and classification tasks.

**TABLE 2:** Classifier performance as a function of feature set and classification performance

Metric	Feature	NT1 versus control	NT1 versus NT2	NT1 versus IH	NT1 versus IH + NT2	NT1 versus All
AUC_ROC	Whole‐night sleep metrics	*0.949* (*0.03*)	0.841 (0.04)	*0.914* (*0.03*)	*0.886* (*0.03*)	*0.891* (*0.03*)
	Sleep metrics, quarter‐night	0.89 (0.05)	0.850 (0.04)	0.896 (0.03)	0.880 (0.03)	0.870 (0.03)
	Transitions, quarter‐night	0.856 (0.05)	*0.856* (*0.04*)	0.888 (0.04)	0.883 (0.03)	0.877 (0.03)
	qEEG, quarter‐night	0.838 (0.07)	0.764 (0.05)	0.862 (0.05)	0.821 (0.04)	0.826 (0.04)
	Hypnodensity, quarter‐night	0.859 (0.05)	0.799 (0.05)	0.836 (0.04)	0.815 (0.04)	0.820 (0.04)
	All features	**0.963 (0.03)**	**0.893 (0.03)**	**0.941 (0.03)**	**0.909 (0.03)**	**0.912 (0.03)**
AUC_PR	Whole‐night sleep metrics	*0.983* (*0.01*)	0.874 (0.04)	*0.922* (*0.04*)	*0.836* (*0.05*)	*0.826* (*0.05*)
	Sleep metrics, quarter‐night	0.956 (0.02)	0.872 (0.04)	0.906 (0.04)	0.811 (0.05)	0.784 (0.06)
	Transitions, quarter‐night	0.945 (0.02)	*0.886* (*0.04*)	0.901 (0.04)	0.839 (0.05)	0.810 (0.06)
	qEEG, quarter‐night	0.936 (0.03)	0.769 (0.06)	0.858 (0.05)	0.691 (0.07)	0.676 (0.08)
	Hypnodensity, quarter‐night	0.947 (0.02)	0.831 (0.05)	0.851 (0.04)	0.744 (0.05)	0.720 (0.06)
	All features	**0.987 (0.01)**	**0.913 (0.03)**	**0.944 (0.03)**	**0.864 (0.04)**	**0.860 (0.05)**
F1	Whole‐night sleep metrics	*0.880* (*0.04*)	0.771 (0.05)	*0.852* (*0.04*)	*0.759* (*0.04*)	*0.733* (*0.04*)
	Sleep metrics, quarter‐night	0.837 (0.04)	0.800 (0.04)	0.820 (0.04)	0.749 (0.04)	0.703 (0.05)
	Transitions, quarter‐night	0.819 (0.04)	*0.814* (*0.05*)	0.817 (0.04)	0.733 (0.05)	0.633 (0.05)
	qEEG, quarter‐night	0.797 (0.06)	0.745 (0.05)	0.785 (0.05)	0.682 (0.05)	0.653 (0.06)
	Hypnodensity, quarter‐night	0.791 (0.04)	0.759 (0.05)	0.756 (0.05)	0.671 (0.05)	0.641 (0.05)
	All features	**0.904 (0.03)**	**0.819 (0.04)**	**0.870 (0.04)**	**0.775 (0.04)**	**0.738 (0.05)**

Classifier performance as a function of feature set and task. Metrics listed are AUC_ROC (area under the curve, ROC), AUC_PR (area under the precision‐recall curve) and F1 score. Mean (SD) of each performance metric is shown, with statistics computed across 200 realizations of each classifier. The best‐performing feature set for each metric and task is shown, highlighting that the combination of all features is best in all cases. The second‐best feature set is denoted with italics; standard whole‐night metrics (which include REM onset) are second‐best performing except for NT1 versus NT2 discrimination. IH, idiopathic hypersomnia; NT1, narcolepsy type 1; NT2, narcolepsy type 2; qEEG, quantitative electroencephalogram.

#### 3.2.1 NT1 versus clinical controls

**FIGURE 3:**
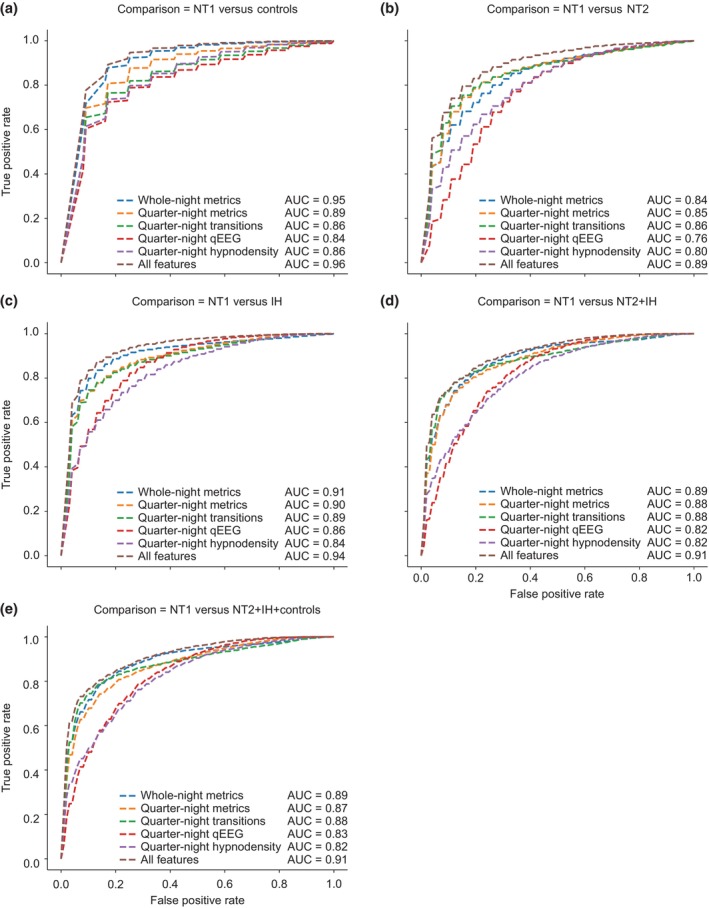
A combination of all features had the best discriminatory importance for all classification tasks. Mean AUC values using the Random Forest (RF) classifier for: (a) NT1 versus control; (b) NT1 versus NT2; (c) NT1 versus IH; (d) NT1 versus NT2 + IH; (e) NT1 versus NT2 + IH + control; (f) AUC values for all comparisons. AUC, area under the receiver operating characteristic curve; IH, idiopathic hypersomnia; NT1, narcolepsy type 1; NT2, narcolepsy type 2; qEEG, quantitative electroencephalogram.

**FIGURE 4:**
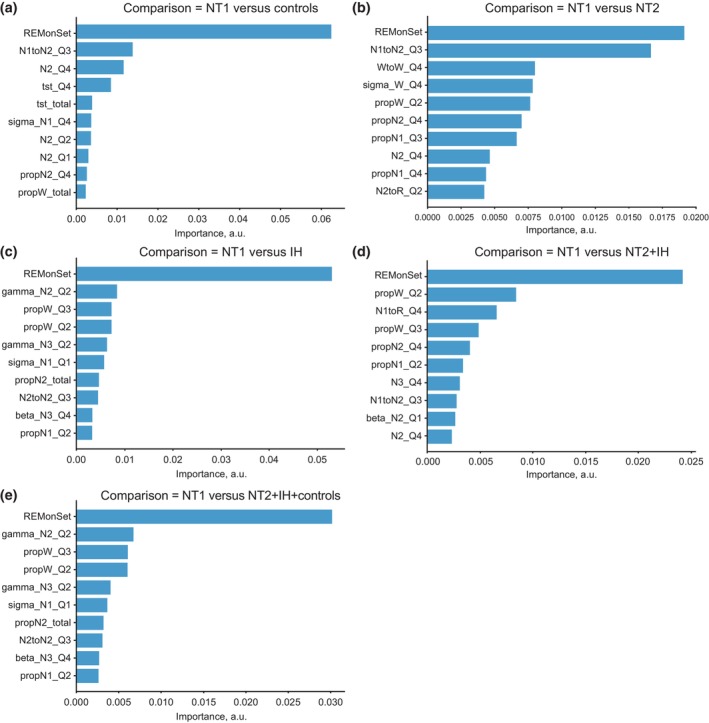
Top 10 highest‐ranked features for each classification task when all features were used as input. Top 10 highest ranked features for: (a) NT1 versus control; (b) NT1 versus NT2; (c) NT1 versus IH; (d) NT1 versus NT2 + IH; and (e) NT1 versus all others. The computed feature importance rank shows the drop in F1 score if the selected feature is removed from the classifier. Absolute levels of feature importance are lower for the comparisons in involving NT2, showing no single feature is critical for classification performance. a.u., arbitrary units; AUC, area under the receiver operating characteristic curve; IH, idiopathic hypersomnia, N1–3, sleep stage N1–3; N2‐Q1, first‐quarter values of fraction of N2; NT1, narcolepsy type 1; NT2, narcolepsy type 2; prop, proportion; Q1–4, quarter 1–4; R, REM sleep; REMonSet, REM sleep‐onset latency; Sigma N1 Q1, first‐quarter values of sigma power during N1 sleep; SSI, stage shift index; TST, total sleep time; W, wake; WR–Q1: wake/REM hypnodensity mixtures. Sleep quantities resolved by quarter of night are denoted as (metric)‐(quarter); e.g. N1–3 is sleep stage N1 in quarter 3 of the night. Time‐resolved hypnodensity mixtures are noted by combining abbreviations for both states, and appending quarter‐night; e.g. WR‐1 denotes Pr(Wake)*Pr(REM) in quarter 1 of the night. Time‐resolved qEEG metrics conditional on sleep state are denoted with band, sleep state and quarter of night; e.g. sigma_N1_Q1 denotes sigma band power during N1 sleep in quarter 1 of the night.

The combination of all features showed the best average AUC_ROC for NT1 versus clinical controls (0.96; Figure 3a), with whole‐night metrics as the second‐best feature set (AUC_ROC 0.945; Figure 3). REM sleep‐onset latency (included in the whole‐night metrics feature set) was found to be the most discriminant feature (Figure 4a). Evaluation of feature importance as a function of classification task showed a large difference between the most important feature (REM sleep‐onset latency; 0.06 arbitrary units [a.u.]) and the second most important feature (N1 to N2 transition in the third quarter‐night; 0.012 a.u.) for the NT1 versus clinical controls comparison (Figure 4a), highlighting the key role of REM sleep‐onset latency in distinguishing NT1 from clinical controls.

#### 3.2.2 NT1 versus other groups

**FIGURE 5:**
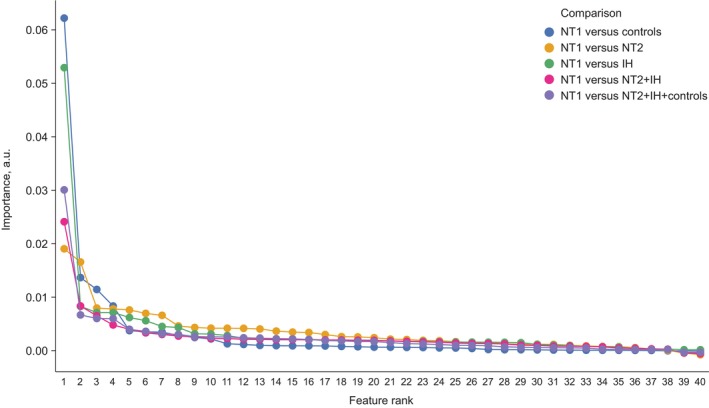
Comparison of the feature importance for each classification task plotted as a function of the final number of features used by the classifier, after elimination of redundant features. All features plotted are greater than zero (though for NT1 versus IH, and NT1 versus NT2, the values are very small). Adding the NT2 cohort decreases the difference of importance between the first and second most important features. a.u., arbitrary units; IH, idiopathic hypersomnia; NT1, narcolepsy type 1; NT2, narcolepsy type 2.

For NT1 versus NT2, NT1 versus IH, NT1 versus NT2 + IH, and NT1 versus All (Figure 3b–f; Table 2), the combination of all feature sets showed the best average AUC_ROC (0.89–0.94; Figure 3 and Table 2). Whole‐night metrics was the second‐best feature set for NT1 versus IH and NT1 versus NT2 + IH comparisons (AUC 0.89–0.91; Table 2). In contrast, quarter‐night transitions were the second‐best feature sets for the NT1 versus NT2 task (AUC_ROC 0.86; Table 2), closely followed by quarter‐night sleep metrics. For NT1 versus NT2, the only early night feature found to be important was REM sleep onset, although its importance is almost tied with the second strongest feature (Figure 4). For NT1 versus IH, the qEEG features appear particularly informative, accounting for four of the top 10 features (Figure 4c), versus 1 for NT1 versus clinical controls or NT1 versus NT2. The remaining comparisons that combine subject groups (NT1 versus NT2 + IH and NT1 versus All) unsurprisingly share many of the top features highlighted in the earlier comparisons (Figure 4d,e). As with the NT1 versus clinical control comparison, evaluation of feature importance for NT1 versus IH showed a large difference between the first and second most important features, highlighting a key role of REM sleep‐onset latency in distinguishing NT1 from IH (Figures 4 and 5). However, for the three comparisons that include NT2, there was a more gradual decline in feature importance, indicating that REM onset was not as dominant and that no single feature (or small group of features) differentiated these groups in our dataset (Figures 4 and 5). Thus, the final discriminating power of the classifier comes from the sum of many small contributions of individual features.

## 4 DISCUSSION

Our results confirm that changes in sleep architecture on nocturnal PSG evaluated using machine learning models could distinguish NT1 from clinical controls, NT2 and IH (mostly the long sleep phenotype). Using machine learning, we found that a combination of multiple feature sets capturing different aspects of sleep, rather than any individual feature set, best distinguished NT1 from these populations and highlighted the importance of time‐dependent changes in sleep.

Few studies have attempted to fully characterize temporal sleep dynamics (Hermans et al., 2022), although sleep characteristics are known to vary over the course of the night. Indeed, the duration of REM sleep episodes typically increases during each cycle, and both the percentage and duration of REM sleep decrease with age. In healthy individuals, REM sleep accounts for approximately 20%–25% of sleep with an average latency of 90–100 min (Carskadon & Dement, 2011; Maski et al., 2020), while mean REM latency is approximately 30 min after sleep onset in patients with NT1 (Zhang et al., 2021). The timing of REM sleep reflects an interaction between the circadian pacemaker and a homeostatic process; whilst timing is more dependent on circadian phase than duration of previous wakefulness, the converse is true for depth of non‐REM sleep (Wurts & Edgar, 2000). One component of our main hypothesis was that resolving sleep by quarter‐night segments would lead to better characterization of DNS in NT1, and better discrimination between NT1, clinical controls, NT2 and IH. Our findings confirm this hypothesis, with differences between individuals with NT1 and clinical controls often being clearest in the first quarter‐night. These findings on the value of dividing sleep by quarter‐night periods are consistent with a recent study (Cesari et al., 2022). In addition to REM sleep, multiple other sleep features had significant interactions between quarter‐night and diagnosis, showing that the time resolution harbours useful diagnostic information.

In addition to time‐resolving sleep, we computed multiple feature sets that captured different aspects of sleep data (standard features such as proportion of time spent in each state, sleep state transition matrices, qEEG and hypnodensity metrics designed to capture mixed sleep states). Several of these features (sleep‐state transition probabilities and mixed‐state probabilities from hypnodensities) reflect sleep instability, known to be important in narcolepsy. Two important findings are that, for all classification problems considered, (a) the combination of all features consistently outperformed individual groups of features, and (b) the top‐ranked features originated from all feature sets. This shows the value of a multi‐faceted approach to quantifying nocturnal sleep in patients with narcolepsy and IH. Our finding that multiple features are helpful for classification are consistent with a previous study showing that the optimal number of features ranged between 65 and 100 depending on the classification problem (Cesari et al., 2022).

Our work builds on findings from Stephansen et al. (2018), who introduced the concept of the hypnodensity approach to sleep medicine. Hypnodensities estimate the probability of each of five possible sleep states assigned to each 30‐s epoch rather than labelling the epoch in a categorical fashion as belonging only to the highest‐probability sleep state. Using this approach, potential mixed sleep and wake states (proposed as a marker of NT1) are captured by pairwise products of the state probabilities, an approach also leveraged here. Unlike our approach, Stephansen and colleagues grouped individuals with NT2 and IH together into an “other hypersomnolence” group and did not include CSF orexin measurements (Stephansen et al., 2018); in addition, the clinical interpretation of some of the statistical features used to describe hypnodensities is challenging. Our work focused on the most clinically interpretable hypnodensity features, that is, averages of state probabilities or their products. Although some degree of performance may be sacrificed by focusing on more interpretable features, our classifier performance in the validation set (0.96 for NT1 versus clinical controls, and 0.94 for NT1 versus IH) was comparable to values reported by Stephansen et al. (AUC 0.94 for high‐pretest or 0.96 for the replication set for closest comparisons; Stephansen et al., 2018). Additionally, Stephansen et al. showed a substantial increase in NT1 detection performance by including HLA phenotyping (Stephansen et al., 2018), whereas here we focus on using only PSG data. Nevertheless, our work replicates their finding that wake–REM and N1–REM mixtures (hypnodensity products) can be important features in NT1 earlier in the night (with higher mixture values in NT1).

The recent study by Cesari et al., which used classifiers trained on multiple feature sets resolved by quarter‐night, had several important differences from our work, including a smaller sample size (40 NT1 individuals, 26 NT2, 23 IH, and 54 with subjective sleepiness), drug intake was permitted (25 with stimulants and/or antidepressants versus drug‐free participants only in our study), and no CSF orexin measurement was included (Cesari et al., 2022). In addition, there were several differences in feature sets; Cesari et al. (2022) did not include qEEG features, but they included additional features utilized by a recently developed open‐source, validated sleep‐scoring algorithm designed to offer high sleep‐staging accuracy (YASA algorithm; Vallat & Walker, 2021) as well as additional sleep bout information. Importantly, Cesari et al. (2022) employed the original, less interpretable features from Stephansen et al. (2018), with the result that top‐ranked features may be difficult to interpret clinically; for example, they found the most important feature for discriminating NT1 from other diagnoses was the time taken before 5% of the sum of the product between N2 and REM, calculated at every epoch, has accumulated, weighed by the total amount of this sum. This suggests that these more complex metrics, while difficult to interpret clinically, may be of real value in diagnosis. In contrast, our study highlights salient PSG features, for example, early REM sleep, time‐dependent changes in sleep changes, qEEG and mixed sleep and wake states, which are selected for interpretability and could aid in patient diagnosis being clinically relevant to differentiate between NT1, clinical controls, and NT2, and IH.

We computed hypnogram‐based metrics from manual scoring, rather than employing algorithmic scoring. We also computed hypnograms using the Stanford Stages automated sleep scoring code (Stephansen et al., 2018). While this algorithm has shown good performance versus human raters (Cesari et al., 2021), we observed a small number of cases in which the automated scoring incorrectly detected early REM epochs. Given the importance of early REM in diagnosis, we anticipated this would lead to lower‐quality input features. Because we are seeking to use machine learning as a tool to characterize important features of central hypersomnolence disorders, we chose to use the highest quality hypnograms available to us, which were the manually scored hypnograms. This suggests that if automated scoring is an input for NT1 detection, it might be useful to weight model errors during training to penalize incorrect scoring of early REM. As automated sleep scoring systems are advancing rapidly, we anticipate future algorithms will have improved accuracy.

Our study provides a key methodological contribution to the evaluation of PSG architecture and continuity in narcolepsy and IH. We describe a machine learning analysis framework to stabilize performance under class imbalance and limited data size, using resampling to yield a more realistic estimate of performance on unseen data (Zhang et al., 2022). This is important because, although the dataset analysed here is large within the context of the relatively low‐incidence central hypersomnolence disorders (collected over many years in a National Reference Center for narcolepsy), many machine learning methods assume the existence of much larger datasets (thousands of samples), which is challenging in disorders with low prevalence. Characteristics of the dataset such as class imbalance, correlated features and the limited dataset size imposed significant challenges in the design of a robust classification strategy. Class imbalance was addressed by using synthetic minority over‐sampling technique (SMOTE)‐based data augmentation (Chawla et al., 2002); tests without SMOTE‐based data augmentation resulted in significantly biased classifiers. Correlated features were identified and removed using a recursive feature method. In early testing we used a single data split, tuning and training the classifiers on 70% of the data and testing on the remaining 30%. However, this strategy could lead to splits in which the performance on the 30% testing set might not be representative due to the relatively small number of individuals in each class (especially clinical controls). Thus, we added a nested cross‐validation, repeating the 70/30 split 200 times and collecting performance statistics across each realization. This approach allowed us to form a more confident estimate of classifier performance and to estimate an average feature importance more robustly. The importance of each feature was computed, showing the drop in AUC on removal of that feature from the classifier. We report the distributions of AUCs across various random splits, demonstrating that performance varied depending on the random split. In addition, we compared training/validation versus the final reported test set results to verify the model is not overfitting (Table S3).

The machine learning results replicated earlier findings that REM sleep‐onset latency is a key feature of NT1 (Um et al., 2022; Zhang et al., 2018). In part because REM sleep‐onset latency is such a dominant feature, the classification performance gains from adding new features were somewhat limited when classifying NT1 versus clinical controls. However, when given the opportunity to select between both time‐resolved and whole‐night features, the machine learning method generally selected time‐resolved features, and the combination of all feature sets provided the best performance in all tasks. Altogether, our result suggests that the classifier can exploit combinations of features to improve performance, rather than finding individual features that separate the two groups.

Our study had several strengths: participants were well‐characterized, all participants were drug‐free, and diagnoses of NT1, NT2 and IH were confirmed through standardized clinical interview, PSG‐MSLT, long PSG recordings for IH patients with long sleep time, and CSF orexin measurements when possible. All participants were diagnosed in the same laboratory and thus consistent diagnostic criteria were applied, especially important when diagnosing NT2 and IH. A corresponding weakness of analysing data from a single site is that the dataset is smaller, and our conclusions may thus be less generalizable than those in multi‐site studies. Another limitation is the control group is small, predominantly female, and consists of clinical controls rather than healthy controls, that is, with a daytime sleepiness without objective hypersomnolence disorder. Confirming our findings in a large multi‐site dataset is an important next step and, in future work, we plan to apply the analysis framework presented here to better understand NT2 and IH, for example, by identifying features that separate NT2 with normal versus intermediate CSF orexin‐A concentrations, and IH with and without long sleep time, and features that separate NT2 or IH from clinical controls. Due to the large number of analyses currently reported and the lack of inclusion of IH patients without long sleep time, such latter comparisons were not done for the present study focusing on NT1.

These results provide a key methodological contribution to the evaluation of PSG architecture in NT1, by highlighting salient PSG features and the relevance of assessing their time‐dependent changes during sleep that could aid in patient diagnosis and differentiation between NT1, NT2 and IH. These features could be assessed in clinical studies to evaluate the impact of novel therapeutics on disease‐specific features of sleep.

## AUTHOR CONTRIBUTIONS

**Marco Vilela:** Writing – original draft; writing – review and editing; formal analysis; conceptualization; methodology. **Brian Tracey:** Writing – review and editing; formal analysis. **Dmitri Volfson:** Writing – review and editing; formal analysis; conceptualization; methodology. **Lucie Barateau:** Writing – review and editing; investigation; conceptualization; methodology. **Alice Cai:** Writing – review and editing; formal analysis. **Derek L. Buhl:** Writing – review and editing; conceptualization; methodology. **Yves Dauvilliers:** Writing – review and editing; investigation; conceptualization; methodology.

## FUNDING INFORMATION

Funding for this work was provided by Takeda Development Center Americas, Inc. and Appel d'Offres Interne, SOMNOBANK CHU Montpellier‐France. Editorial assistance in formatting, proofreading, copy editing and fact‐checking was provided by Excel Medical Affairs, and was funded by Takeda Development Center Americas, Inc.

## CONFLICT OF INTEREST STATEMENT

Lucie Barateau received funds for travelling to conferences by Idorsia and Bioprojet, and board engagements by Jazz, Takeda, Idorsia and Bioprojet. Yves Dauvilliers received funds for seminars, board engagements and travel to conferences by Jazz, Orexia, Idorsia, Takeda, Avadel and Bioprojet. Marco Vilela, Brian Tracey, Dmitri Volfson, Alice Cai and Derek L Buhl are or were employees of Takeda Development Center Americas, Inc., and stockholders of Takeda Pharmaceutical Company Ltd at the time of this study.

## Supporting information


**DATA S1.** Supporting Information

## Data Availability

The datasets supporting this analysis will be made available from the corresponding author on reasonable request.
