# Supplementary material for: Identifying time‐resolved features of nocturnal sleep characteristics of narcolepsy using machine learning
Source: J Sleep Res. 2024 Apr 26;33(6):e14216. doi: 10.1111/jsr.14216 (PMC11596988; doi:10.1111/jsr.14216)
Supplement: Supplementary file 1 — DATA S1. Supporting Information [file JSR-33-e14216-s001.docx]

# SUPPLEMENTARY MATERIAL

**Identifying time-resolved features of nocturnal sleep characteristics of narcolepsy using machine learning**

Marco Vilela^1^, Brian Tracey^1^, Dmitri Volfson^1^, Lucie Barateau,^2^ Alice Cai^1^, Derek L. Buhl^1*^, Yves Dauvilliers^2^

^1^ Takeda Development Center Americas, Inc., Cambridge, Massachusetts, USA

^2^Sleep-Wake Disorders Center, Department of Neurology, Gui-de-Chauliac Hospital, CHU Montpellier; National Reference Network for Narcolepsy, Montpellier, France; Institute for Neurosciences of Montpellier (INM), INSERM, University of Montpellier, France

## Supplementary methods

### **Classifier framework**

**Inner loop:** Within the inner loop, participants were randomly divided into a train/validation set (70% of participants) and a test set (30% of participants). Stratified sampling was used to ensure the distribution of subject diagnoses in these datasets mimicked that in the overall dataset. The train/validation set was used for feature selection and hyperparameter optimization, while the test set was used for computation of performance metrics.

Depending on the classification task (NT1 vs. controls, NT1 vs. NT2, etc.) varying degrees of class imbalance were present in the data (worst case: 114 NT1 vs. 41 clinical controls). The impact of class imbalance in the training/validation dataset was mitigated using the synthetic minority over-sampling technique (SMOTE) data augmentation algorithm, which synthesizes new data points for the minority class based on the real data (Chawla, Bowyer, Hall, & Kegelmeyer, 2002) (note that SMOTE was not applied to test data). After data augmentation, a two-step feature selection process (combined in ‘Feature Selection’ in Figure 1) was applied. First, features with low variance among the classes were removed (lower than 10^-10^) and missing values (approximately 1% of data) were imputed using the average of the entire dataset. Second, the remaining features were fed into a recursive feature elimination method (Guyon, Weston, Barnhill, & Vapnik, 2002) using a XGBoost classifier model (Friedman, 2002). This feature elimination removed the lowest ranked feature at each iteration, where the feature rank is estimated based on the classifier’s optimization function mean value out of a 10-fold cross-validation, with the F1 score used as the XGBoost cost function. XGBoost was chosen for this task because of its stability in estimating feature rank.

After performance of the above steps, the remaining features were used to optimize hyper-parameters of classification models in a 10-fold, 10 repeat cross-validation loop (Figure 1). The hyperparameters optimized were: maximum tree depth, number of trees and maximum number of features per tree for random forest, kernel function for Gaussian process, maximum tree depth, number of trees and learning rate for theXGBoost. The F1 score was used to optimize all three classifier hyper-parameters on the train/validation set and it was selected as the main metric of performance in all classification tasks. The optimal hyper-parameters and selected feature set was used to retrain the classification model on the combined train and validation set (“Train optimized classifier” box in Fig. 1), and the performance of this model was evaluated using the test set. As part of this process, model performance (both on the validation set used for hyperparameter tuning, and on the test set) were stored for later analysis.

**Outer loop**: Classifier final performance can show some variability depending on the random split of the data between the train/validation and test sets (Hawkins, Basak, & Mills, 2003; Kuhn & Johnson, 2013). In the feature space, data points located where the distribution of the classes overlap are harder to correctly classify than points that are away from the possible decision boundary between classes, which characterizes the Bayes error rate (Chen, Cao, Xing, & Liang, 2023). Because of the limited sample size, points near or at the classes' decision boundaries have a much higher impact on the average performance metric. We mitigate this problem by resampling the data in an outer loop using Monte Carlo resampling (Kuhn & Johnson, 2013). Thus, the inner loop described in Figure 1 was repeated 200 times for each classification task with different randomly selected stratified data splits, such that mean and standard deviation values for all metrics reported could be estimated. This approach not only removes the subjectiveness of choosing one F1 value associated with a specific data split, but it also gives a measure of classification variability that can help plan application of the classifier.

**Performance statistics and feature importance**: Tabulated model performances for both the training/validation data (found during hyperparameter tuning) and the testing data were computed and compared to check for overfitting. For each inner loop classifier model, the importance of each feature was estimated by measuring the impact of randomizing the feature’s values (via shuffling) on the classifier cost function (Altmann, Tolosi, Sander, & Lengauer, 2010). Shuffling an important feature should result in a significant drop in the cost function while no significant reduction should be observed for unimportant features. Using this approach, we listed the top features, then sorted by feature importance to determine whether classification performance is dominated by a few strong features or by a combination of many weaker features. Feature importance values computed during the 200 inner loops were then averaged to produce an estimated average feature importance.

### **Mixed model analysis: methods and results**

**Methods:**
Models were fit using lmer for each endpoint, capturing age, quarter-night, diagnosis, and the interaction between quarter-night and diagnosis, with random offsets for each participant. Prior to mixed model analysis, a Shapiro test was performed to determine whether a variable transformation (log or square root) would make the endpoint more normally distributed; if it did, the transformation was applied. P-values for diagnosis and diagnosis-by-quarter-night interaction were extracted from ANOVA analysis of each mixed model, and a Benjamini-Hochberg false discovery rate correction was used to adjust p-values to account for multiple comparisons. Significant diagnosis-by-quarter-night interactions indicate that the time evolution of the sleep feature varies by diagnosis.

**Results**:
Features with significant quarter-night by diagnosis interactions are summarized in Suppl Table 7. Significant diagnosis-by-quarter-night interactions were found for the proportion of time in sleep states, additional whole-night and quarter-night sleep metrics (Suppl Figure 1), several quarter-night sleep state transitions (W->W, W->N2, W->R, N1->N1, N2->N2, N2->N3, N2->R, N3->W, N3->N3, and R->R; Suppl Figure 2), key qEEG band powers (delta, theta, alpha, sigma in Wake, N1, N2, and N3; Suppl Figures 3-7), and several mixed probabilities (W*N1, W*R, N1*N2, N1*R, N2*N3; Suppl Figure 8 ). Differences in within-state transition probabilities suggest the groups have variation in sleep and wake bout lengths.

## Supplementary figures


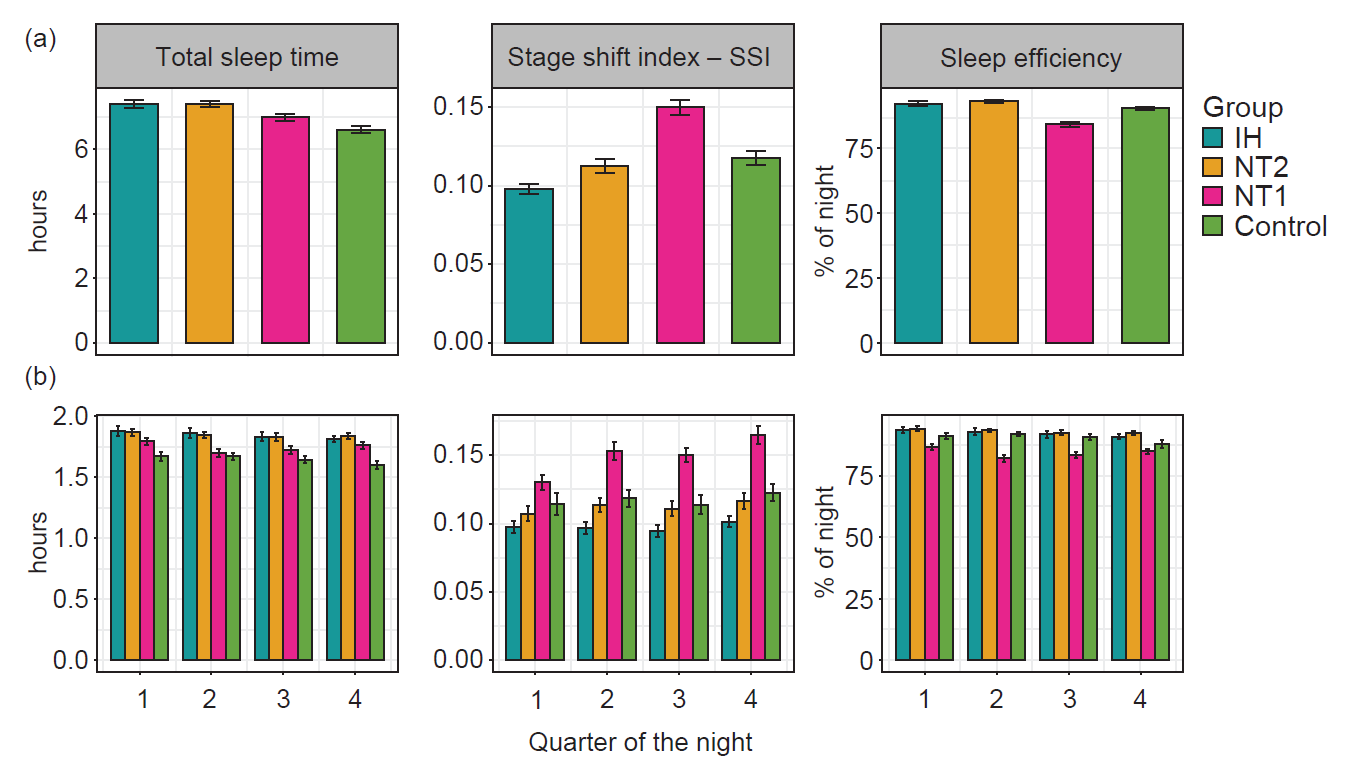


**Suppl Figure 1: Differences between NT1 vs NT2, IH and clinical controls in Whole night and Quarter night sleep metrics**

Total sleep time (hours), stage shift index score, and sleep efficiency (percent of night) computed for whole night (top row) and quarter night (bottom row) periods for each group (NT1, NT2, IH, clinical control). ANOVA analysis results are shown in Suppl Table 1 (whole night sleep metrics) and Suppl Table 2 (quarter-night sleep metrics). Bar plots show the mean and standard deviation. IH, idiopathic hypersomnia; NT1, narcolepsy type 1; NT2, narcolepsy type 2; SSI, stage shift index.


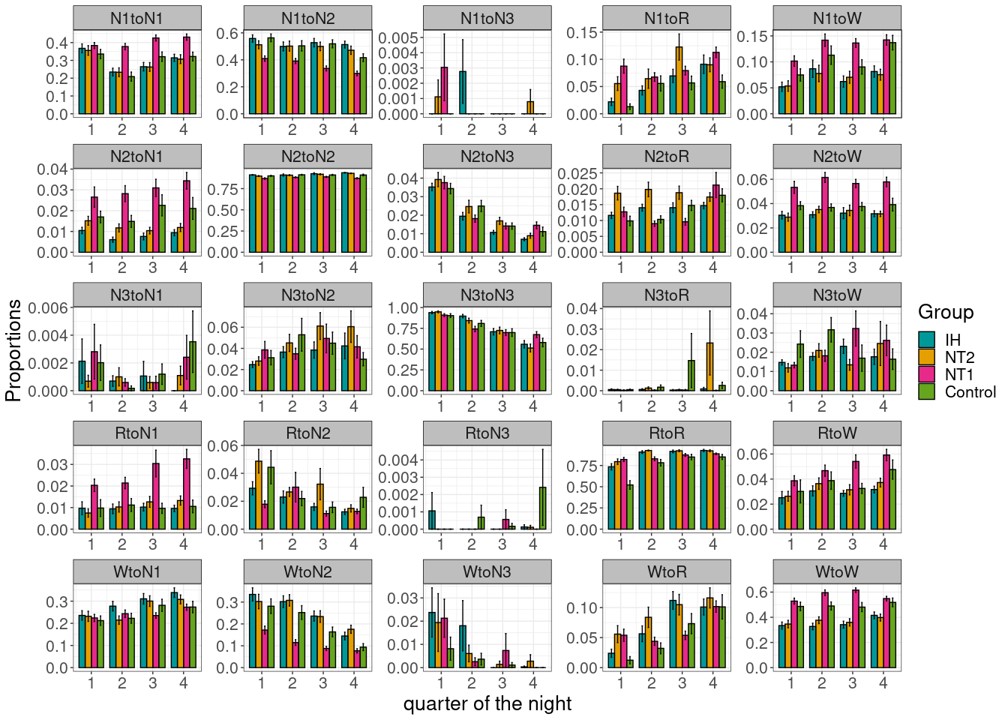


**Suppl Figure 2: Differences between NT1 vs NT2, IH and clinical controls in Quarter night stage transitions**

Sleep stage transitions at the epoch level computed for each quarter night period and expressed as the proportion of total transitions. Transition probabilities for N2-W and N1-N1 were significantly higher in at least 3 of 4 quarter-night periods for patients with NT1 vs NT2, IH and controls, and transition probabilities for W-N2 and N1-N2 were significantly lower. ANOVA results for transition probabilities are shown in Suppl Table 3. Bar plots show the mean and standard deviation. IH, idiopathic hypersomnia; N1-3, sleep stage N1-3; NT1, narcolepsy type 1; NT2, narcolepsy type 2; R, REM sleep; W, wake. Transitions were labeled as: reference sleep stage “to” any given sleep stage. For instance, transitions from N1 to N2 are represented as N1toN2.


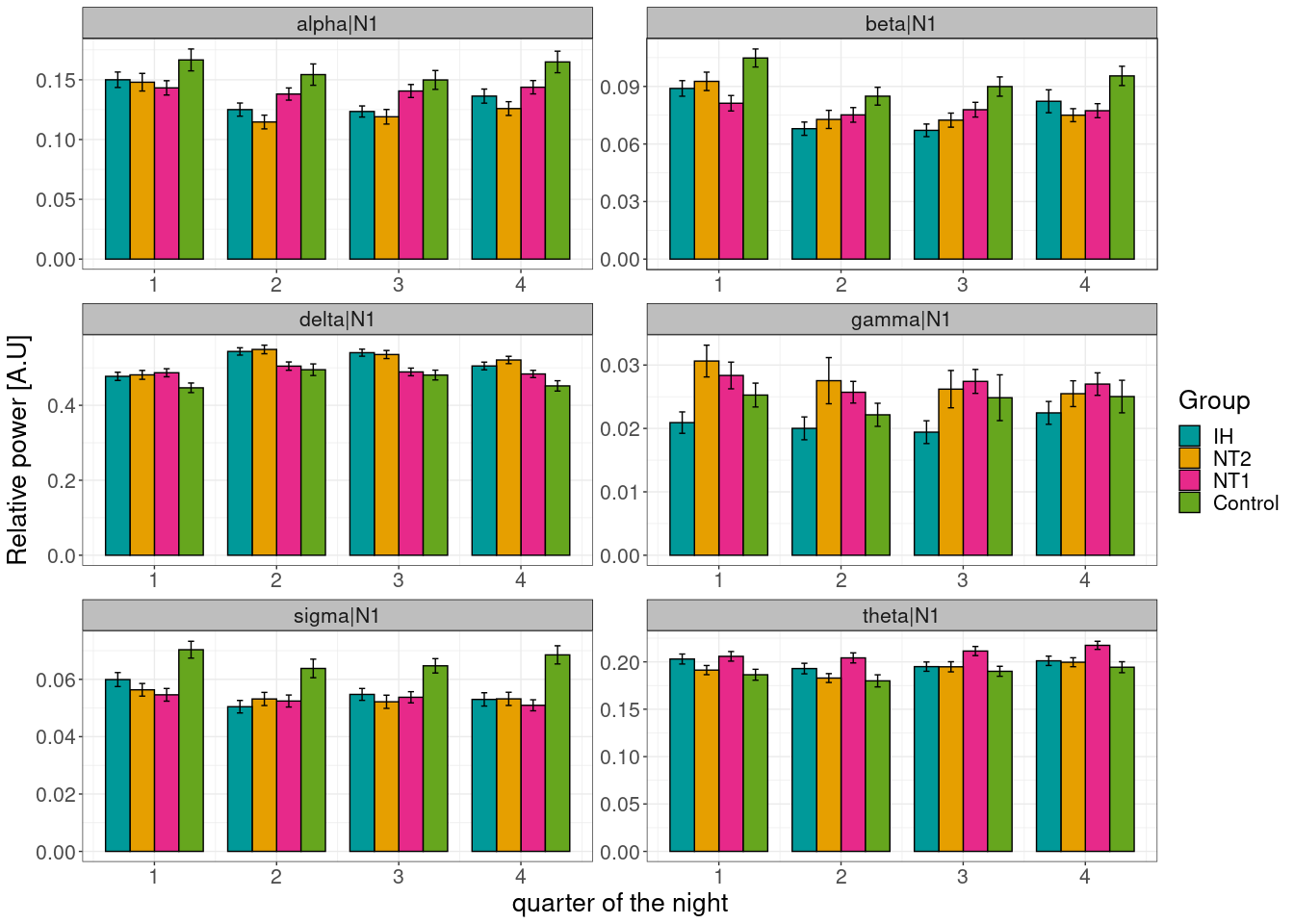


**Suppl Figure 3. Differences between NT1 vs NT2, IH, and clinical controls in qEEG conditional on N1 sleep**

qEEG features computed for each quarter-night period. ANOVA results are shown in Suppl Table 4.

A.U., arbitrary units; IH, idiopathic hypersomnia; N1, sleep stage N1; NT1, narcolepsy type 1; NT2, narcolepsy type 2; qEEG, quantitative Electroencephalogram.


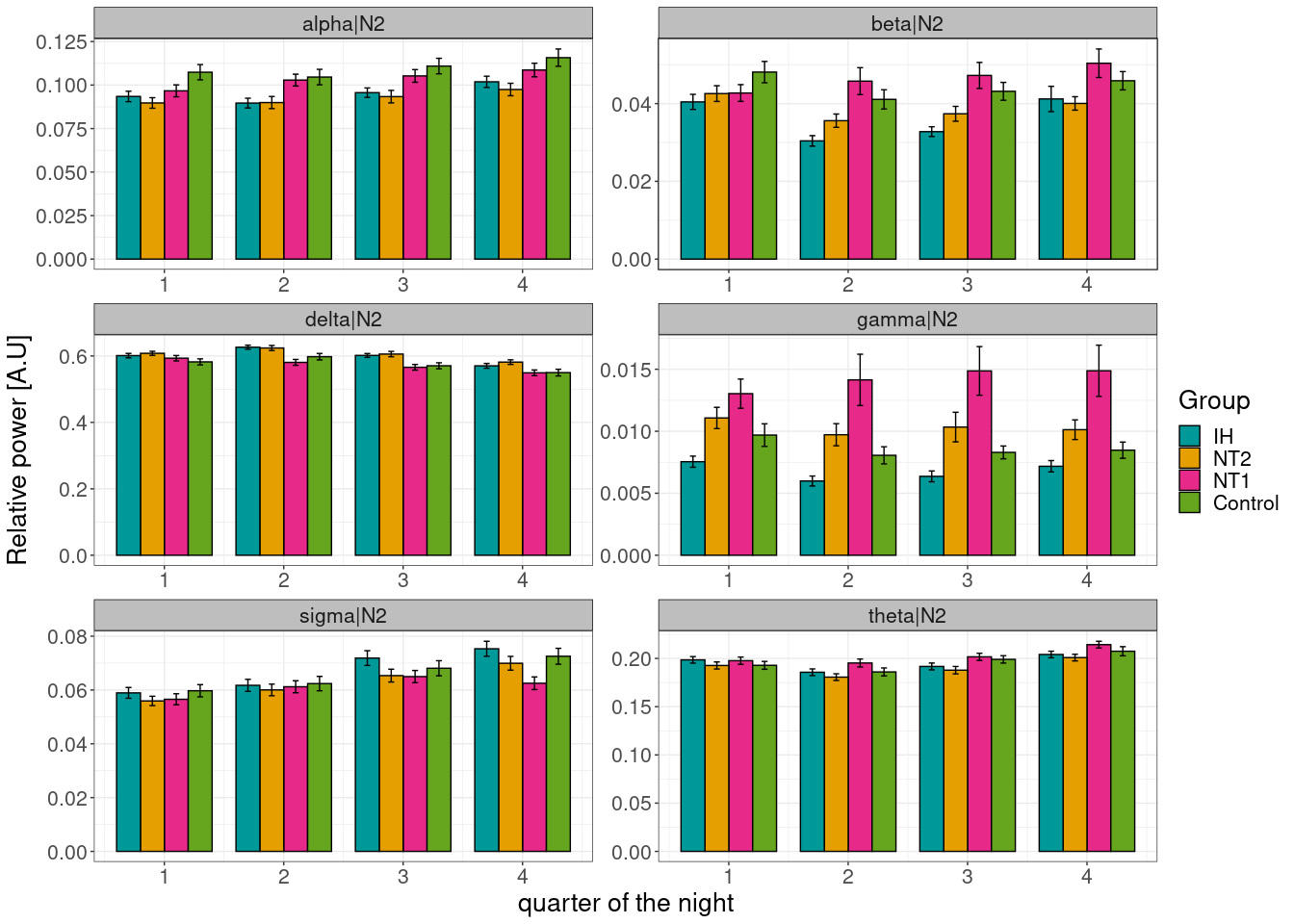


**Suppl Figure 4. Differences between NT1 vs NT2, IH, and clinical controls in qEEG conditional on N2 sleep**

qEEG features computed for each quarter-night period. ANOVA results are shown in Suppl Table 4.

A.U., arbitrary units; IH, idiopathic hypersomnia; N2, sleep stage N2; NT1, narcolepsy type 1; NT2, narcolepsy type 2; qEEG, quantitative Electroencephalogram.


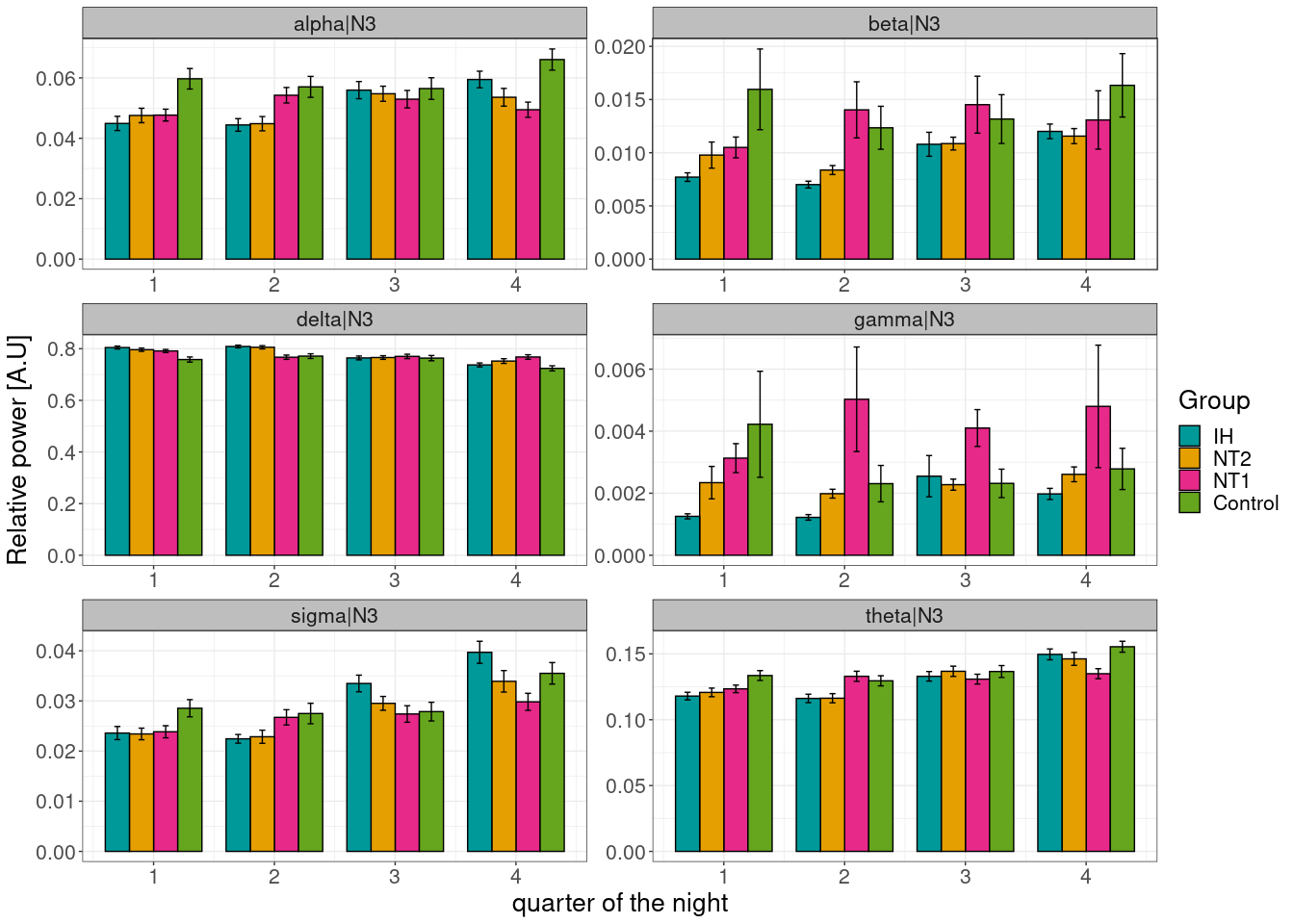


**Suppl Figure 5. Differences between NT1 vs NT2, IH, and clinical controls in qEEG conditional on N3 sleep**

qEEG features computed for each quarter-night period. ANOVA results are shown in Suppl Table 4.

A.U., arbitrary units; IH, idiopathic hypersomnia; N3, sleep stage N3; NT1, narcolepsy type 1; NT2, narcolepsy type 2; qEEG, quantitative Electroencephalogram.


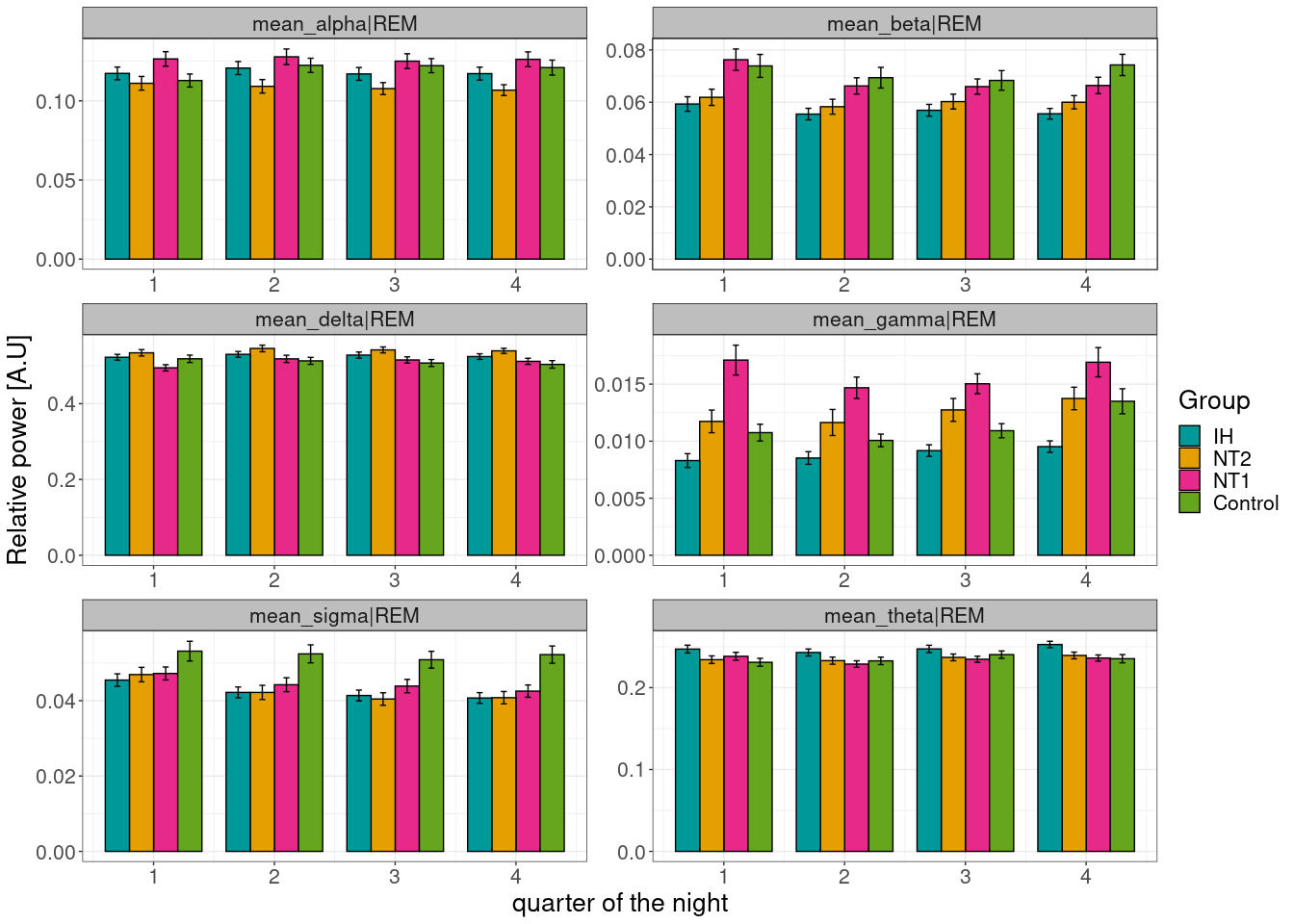


**Suppl Figure 6. Differences between NT1 vs NT2, IH, and clinical controls in qEEG conditional on REM sleep**

qEEG features computed for each quarter-night period. ANOVA results are shown in Suppl Table 4.

A.U., arbitrary units; IH, idiopathic hypersomnia; NT1, narcolepsy type 1; NT2, narcolepsy type 2; qEEG, quantitative Electroencephalogram; REM, rapid eye movement.


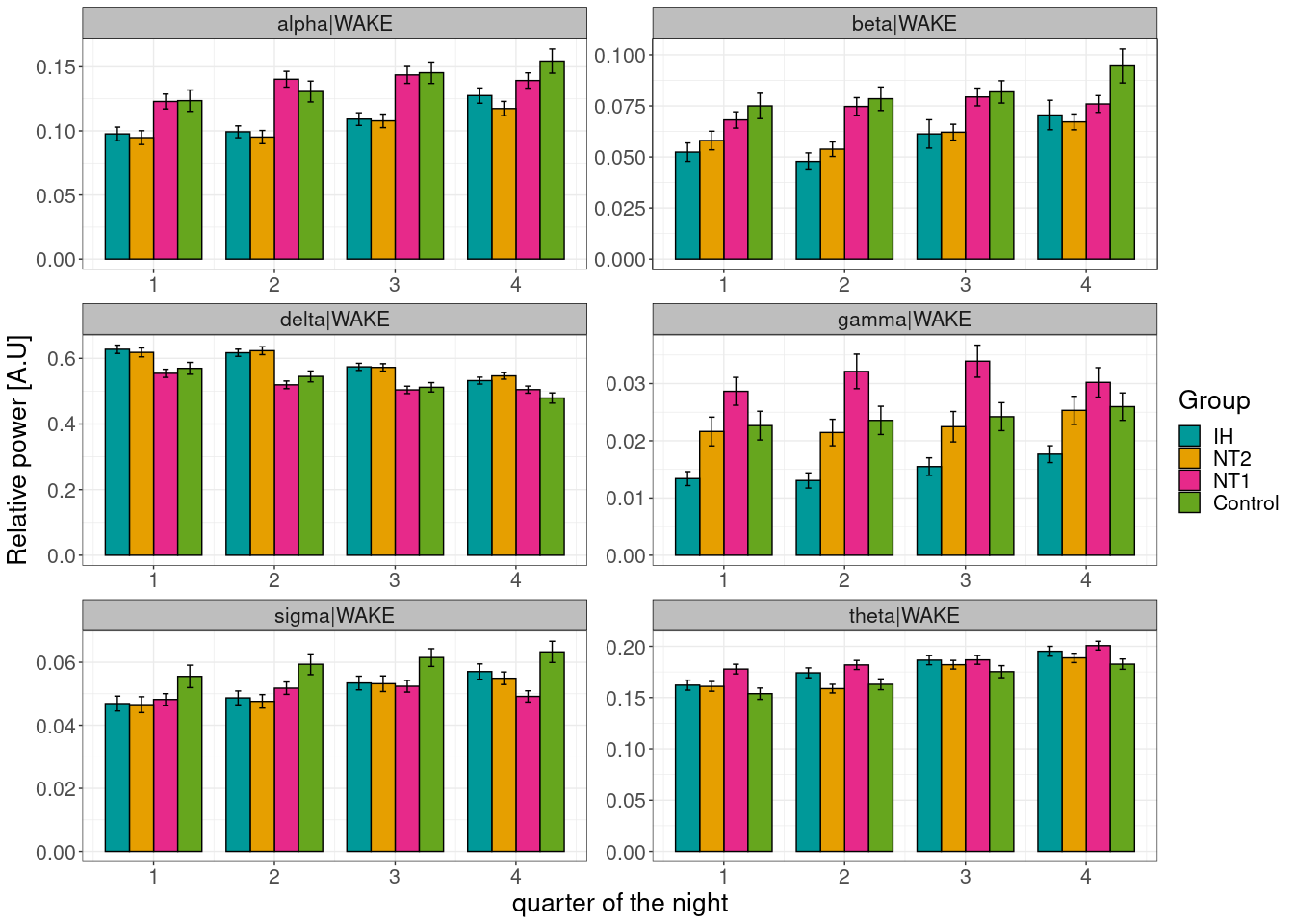


**Suppl Figure 7. Differences between NT1 vs NT2, IH, and clinical controls in qEEG conditional on Wake stage**

qEEG features computed for each quarter-night period. ANOVA results are shown in Suppl Table 4.

A.U., arbitrary units; IH, idiopathic hypersomnia; NT1, narcolepsy type 1; NT2, narcolepsy type 2; qEEG, quantitative Electroencephalogram.


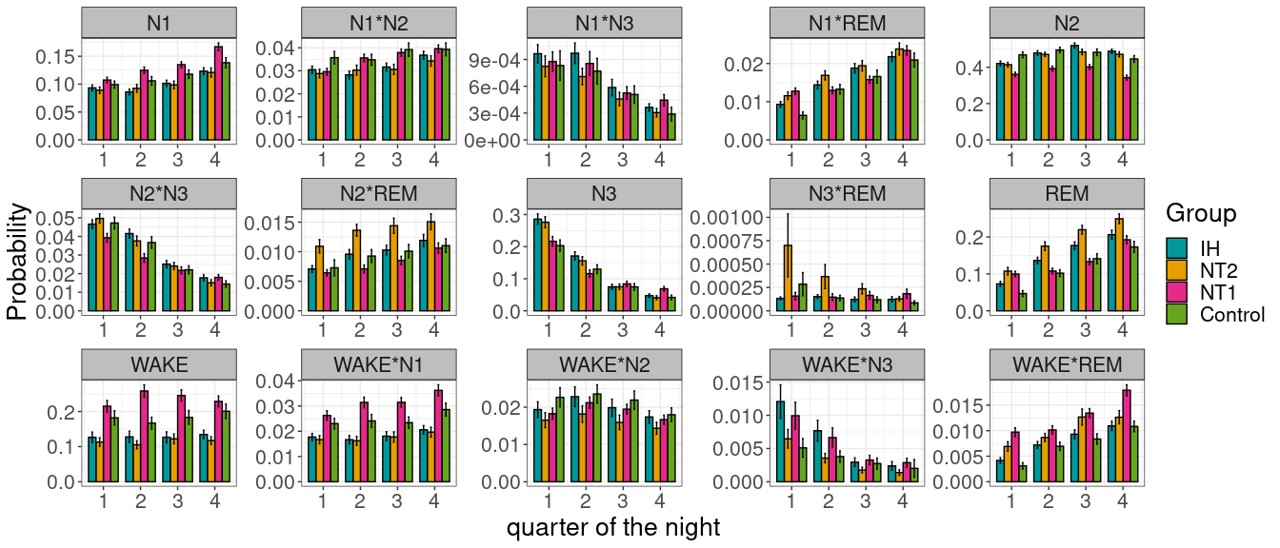


**Suppl Figure 8: Differences between NT1 vs NT2, IH, and clinical controls in Quarter night sleep stage probabilities – Hypnodensity**

Sleep stage probabilities computed for each quarter night period. The probability of each sleep state or mixed sleep state was assigned to each quarter-night period. ANOVA results for quarter-night hypnodensity features are shown in Suppl Table 5.

IH, idiopathic hypersomnia; N1-3, sleep stage N1-3; NT1, narcolepsy type 1; NT2, narcolepsy type 2; REM, rapid eye movement.


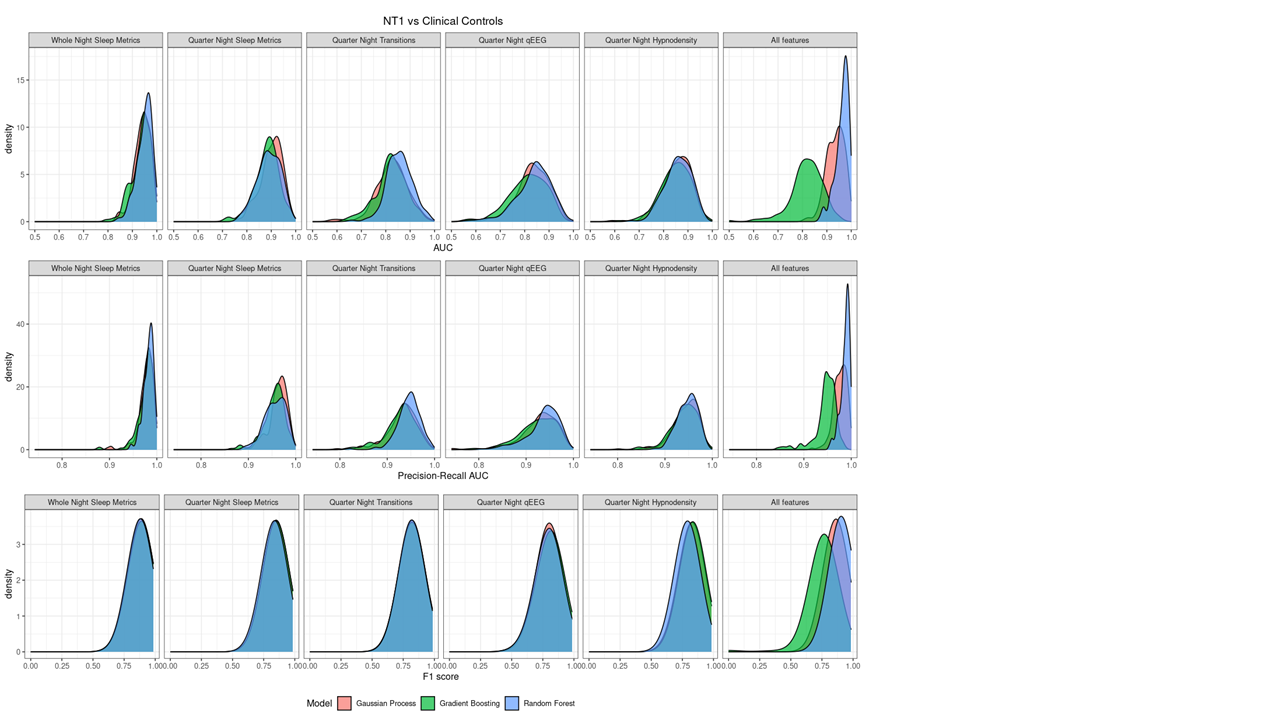


**Suppl Figure 9: AUC, Precision-Recall AUC and F1 for NT1 vs clinical controls**

AUC, sensitivity, and specificity for three classifiers, Gaussian Process, Gradient Boosting (XGBoost) and Random Forest, evaluated in the NT1 vs controls task. The Random Forest classifier was selected as the best performing classifier based on AUC.

AUC, area under the receiver operating characteristic curve; NT1, narcolepsy type 1; qEEG, quantitative Electroencephalogram.

## Supplementary tables

**Suppl Table 1: Mean (SD) values of apnea-hypopnea index (AHI) and periodic leg motion index (PLMI) by patient diagnosis**

|  | Control | NT1 | NT2 | IH |
| --- | --- | --- | --- | --- |
| TST, hours | 6.66 (0.8) | 6.99 (1.1) | 7.4 (0.9) | 7.41 (1.3) |
| Sleep Efficiency, % | 90.7 (3.9) | 84.18 (10.5) | 93.07 (5.5) | 92.2 (10.3) |
| WASO, minutes | 41.7 (19.3) | 78.1 (52.3) | 32.2 (24.1) | 37.2 (47.0) |
| % N1 | 4.3 (2.2) | 8.6 (5.7) | 3.8 (2.6) | 3.5 (2.2) |
| % N2 | 49.9 (7.8) | 38.23 (10.5) | 47.71 (8.8) | 49.32 (8.9) |
| % N3 | 18.9 (5.9) | 16.6 (7.3) | 19.0 (6.1) | 19.2 (6.0) |
| % REM | 17.6 (6.) | 20.7 (6.3) | 22.5 (6.2) | 20.2 (5.8) |
| REM onset, minutes | 100.5 (52.8) | 38.3 (60.3) | 61.8 (47.1) | 90.4 (57.9) |
| AHI, events/hour | 2.66 (3.1) | 9.12 (12.6) | 4.92 (6.8) | 3.7 (5.8) |
| PLMI, events/hour | 2.4 (3.4) | 10.63 (20.0) | 3.88 (10.9) | 3.9 (7.7) |

**Suppl Table 2: ANOVA analysis results for whole-night sleep metrics**

| Feature | Interval | ANOVA_pVal | NT1_vs_NT2 | NT1_vs_IH | NT1_vs_Control | NT1_vs_ALL |
| --- | --- | --- | --- | --- | --- | --- |
| tst_total | 1234 | **1.88e-04** | 1.85e-01 | 1.21e-01 | 4.52e-01 | **6.87e-06** |
| ssi_total | 1234 | **1.81e-17** | **2.57e-08** | **3.29e-16** | **2.63e-04** | **1.21e-66** |
| propN1_total | 1234 | **1.23e-22** | **6.40e-15** | **4.98e-18** | **7.97e-09** | **3.79e-97** |
| propN2_total | 1234 | **8.47e-19** | **6.31e-10** | **2.58e-14** | **2.94e-10** | **3.62e-79** |
| propN3_total | 1234 | **1.28e-02** | 2.15e-01 | 1.09e-01 | 4.46e-01 | **4.26e-11** |
| propR_total | 1234 | **4.24e-04** | 2.92e-01 | 1.00e+00 | **3.39e-02** | 7.81e-01 |
| propW_total | 1234 | **4.26e-13** | **3.65e-09** | **3.91e-08** | **8.82e-04** | **1.12e-54** |
| REMonSet | 1234 | **1.28e-12** | **2.78e-02** | **5.68e-10** | **1.64e-08** | **5.25e-38** |

IH, idiopathic hypersomnia; N1-3, sleep stage N1-3; NT1, narcolepsy type 1; NT2, narcolepsy type 2; prop, proportion; R, REM sleep; REMonSet, REM sleep onset sleep; SSI, stage shift index; TST, total sleep time; W, wake

Bold denotes statistical significance at the level of 0.05.

**Suppl Table 3: ANOVA analysis results for quarter-night sleep metrics**

| Feature | Interval | ANOVA_pVal | NT1_vs_NT2 | NT1_vs_IH | NT1_vs_Control | NT1_vs_ALL |
| --- | --- | --- | --- | --- | --- | --- |
| tst | 1 | **1.10e-02** | 1.00e+00 | 7.88e-01 | 5.33e-01 | 1.95e-01 |
| tst | 2 | **3.12e-04** | **4.48e-02** | **9.14e-03** | 1.00e+00 | **1.09e-03** |
| tst | 3 | **1.52e-03** | 4.28e-01 | 2.91e-01 | 7.56e-01 | **3.89e-02** |
| tst | 4 | **2.26e-04** | 5.51e-01 | 1.00e+00 | **2.02e-02** | 3.73e-01 |
| ssi | 1 | **9.85e-05** | **4.45e-02** | **2.06e-04** | 2.57e-01 | **7.92e-06** |
| ssi | 2 | **1.31e-11** | **6.14e-06** | **6.89e-12** | **7.20e-03** | **2.10e-12** |
| ssi | 3 | **6.24e-13** | **6.33e-06** | **3.37e-12** | **1.65e-03** | **6.42e-14** |
| ssi | 4 | **5.73e-15** | **4.09e-08** | **3.06e-14** | **1.00e-03** | **4.88e-16** |
| propN1 | 1 | **1.13e-04** | **4.24e-03** | **1.56e-03** | **2.45e-02** | **2.63e-06** |
| propN1 | 2 | **3.18e-17** | **2.97e-11** | **1.28e-13** | **9.17e-07** | **6.83e-20** |
| propN1 | 3 | **3.66e-21** | **6.17e-15** | **2.96e-17** | **2.39e-08** | **2.48e-24** |
| propN1 | 4 | **1.16e-17** | **1.40e-11** | **4.26e-15** | **1.99e-06** | **3.17e-20** |
| propN2 | 1 | **2.17e-04** | 5.91e-02 | **3.63e-03** | **4.59e-04** | **3.86e-05** |
| propN2 | 2 | **4.90e-05** | 5.41e-02 | **1.46e-03** | **1.24e-03** | **2.87e-06** |
| propN2 | 3 | **4.97e-09** | **1.64e-04** | **3.87e-08** | **4.75e-03** | **1.35e-10** |
| propN2 | 4 | **3.66e-21** | **3.26e-14** | **1.62e-15** | **1.23e-10** | **2.23e-24** |
| propN3 | 1 | **2.23e-04** | **1.80e-02** | **8.78e-03** | 2.52e-01 | **8.22e-06** |
| propN3 | 2 | **1.86e-03** | **1.30e-02** | **1.85e-03** | 2.41e-01 | **1.05e-04** |
| propN3 | 3 | 4.32e-01 | 1.00e+00 | 1.00e+00 | 1.00e+00 | 9.40e-01 |
| propN3 | 4 | **1.88e-03** | **5.18e-03** | **3.12e-02** | 6.38e-02 | **1.13e-04** |
| propR | 1 | **1.84e-05** | 3.78e-01 | **1.67e-03** | 1.20e-04 | **1.19e-05** |
| propR | 2 | **6.59e-03** | **2.96e-02** | 1.00e+00 | 1.00e+00 | **4.30e-02** |
| propR | 3 | **6.70e-03** | **8.41e-03** | 3.94e-01 | 1.00e+00 | **6.54e-03** |
| propR | 4 | 8.06e-02 | 1.00e+00 | 1.00e+00 | 1.31e-01 | 3.44e-01 |
| propW | 1 | **1.77e-05** | **4.28e-04** | **1.51e-03** | 6.51e-02 | **3.66e-07** |
| propW | 2 | **1.80e-11** | **5.59e-08** | **6.01e-08** | **1.37e-04** | **9.64e-14** |
| propW | 3 | **1.96e-07** | **4.71e-05** | **1.15e-04** | **2.69e-02** | **2.92e-09** |
| propW | 4 | **1.86e-06** | **2.02e-05** | **6.65e-04** | 6.30e-01 | **2.62e-07** |

IH, idiopathic hypersomnia; N1-3, sleep stage N1-3; NT1, narcolepsy type 1; NT2, narcolepsy type 2; prop, proportion; R, REM sleep; SSI, stage shift index; TST, total sleep time; W, wake.

Bold denotes statistical significance at the level of 0.05.

**Suppl Table 4 : ANOVA results for quarter-night sleep state transition probabilities**

| Feature | Interval | anova_pVal | NT1_vs_NT2 | NT1_vs_IH | NT1_vs_Control | NT1_vs_ALL |
| --- | --- | --- | --- | --- | --- | --- |
| WtoW | 1 | **9.37e-06** | **3.03e-03** | **4.79e-04** | 1.00e+00 | **2.30e-06** |
| WtoW | 2 | **2.34e-10** | **6.59e-06** | **1.17e-09** | 5.44e-02 | **3.41e-12** |
| WtoW | 3 | **2.94e-12** | **4.39e-08** | **4.99e-10** | 6.07e-02 | **4.14e-14** |
| WtoW | 4 | **6.16e-05** | **1.21e-03** | **4.05e-03** | 1.00e+00 | **1.47e-05** |
| WtoN1 | 1 | 7.99e-01 | 1.00e+00 | 1.00e+00 | 1.00e+00 | 8.74e-01 |
| WtoN1 | 2 | 1.77e-01 | 1.00e+00 | 1.00e+00 | 1.00e+00 | 9.97e-01 |
| WtoN1 | 3 | 1.87e-01 | 5.47e-01 | 2.11e-01 | 1.00e+00 | **2.34e-02** |
| WtoN1 | 4 | 1.72e-01 | 1.00e+00 | 1.23e-01 | 1.00e+00 | **2.77e-02** |
| WtoN2 | 1 | **8.23e-04** | 7.48e-02 | **3.70e-03** | 5.25e-02 | **1.62e-05** |
| WtoN2 | 2 | **3.14e-08** | **8.74e-06** | **7.08e-06** | **5.70e-04** | **6.92e-11** |
| WtoN2 | 3 | **3.48e-07** | **6.14e-05** | **1.65e-05** | 9.01e-02 | **3.45e-09** |
| WtoN2 | 4 | **1.32e-04** | **4.16e-04** | **3.25e-02** | 1.00e+00 | **2.60e-05** |
| WtoN3 | 1 | 7.06e-01 | 1.00e+00 | 1.00e+00 | 1.00e+00 | 6.55e-01 |
| WtoN3 | 2 | 3.82e-01 | 1.00e+00 | 8.56e-01 | 1.00e+00 | 3.22e-01 |
| WtoN3 | 3 | 7.20e-01 | 1.00e+00 | 8.38e-01 | 1.00e+00 | 2.00e-01 |
| WtoN3 | 4 | 5.75e-01 | 1.00e+00 | 1.00e+00 | 1.00e+00 | 4.34e-01 |
| WtoR | 1 | **3.52e-02** | 1.00e+00 | 2.17e-01 | 9.73e-02 | 6.94e-02 |
| WtoR | 2 | 1.80e-01 | 2.06e-01 | 1.00e+00 | 1.00e+00 | 2.57e-01 |
| WtoR | 3 | **6.66e-03** | 5.72e-02 | **1.25e-02** | 1.00e+00 | **1.49e-03** |
| WtoR | 4 | 4.19e-01 | 1.00e+00 | 1.00e+00 | 9.40e-01 | 9.92e-01 |
| N1toW | 1 | **1.00e-03** | **1.11e-02** | **4.74e-03** | **3.22e-02** | **1.59e-05** |
| N1toW | 2 | **1.95e-02** | **2.19e-02** | 5.57e-02 | 2.32e-01 | **7.37e-04** |
| N1toW | 3 | **7.59e-06** | **5.95e-04** | **2.47e-05** | **5.23e-03** | **4.32e-08** |
| N1toW | 4 | **1.47e-05** | **7.05e-05** | **1.72e-04** | 1.00e+00 | **1.18e-06** |
| N1toN1 | 1 | 6.26e-01 | 1.00e+00 | 1.00e+00 | 7.82e-01 | 3.25e-01 |
| N1toN1 | 2 | **1.09e-08** | **2.37e-05** | **1.05e-05** | **3.30e-06** | **4.12e-11** |
| N1toN1 | 3 | **1.08e-08** | **5.68e-07** | **2.16e-07** | **1.46e-02** | **3.75e-11** |
| N1toN1 | 4 | **3.92e-05** | **3.86e-04** | **5.11e-04** | **8.57e-03** | **2.95e-07** |
| N1toN2 | 1 | **1.73e-04** | **4.03e-02** | **2.75e-04** | **9.24e-04** | **8.33e-06** |
| N1toN2 | 2 | **4.94e-03** | **3.18e-02** | **2.61e-02** | **4.65e-02** | **1.22e-04** |
| N1toN2 | 3 | **1.09e-08** | **4.89e-06** | **3.49e-08** | **1.13e-05** | **2.83e-11** |
| N1toN2 | 4 | **1.67e-09** | **5.81e-07** | **1.41e-10** | **5.49e-03** | **7.70e-12** |
| N1toN3 | 1 | 5.00e-01 | 1.00e+00 | 1.00e+00 | 1.00e+00 | 9.29e-02 |
| N1toN3 | 2 | 3.63e-01 | 1.00e+00 | 7.07e-01 | 1.00e+00 | 3.58e-01 |
| N1toN3 | 3 | NaN | NaN | NaN | NaN | NaN |
| N1toN3 | 4 | 5.24e-01 | 1.00e+00 | 1.00e+00 | 1.00e+00 | 4.88e-01 |
| N1toR | 1 | **1.79e-04** | 3.09e-01 | **1.08e-04** | **2.21e-03** | **5.50e-05** |
| N1toR | 2 | 3.37e-01 | 1.00e+00 | 7.61e-01 | 1.00e+00 | 4.58e-01 |
| N1toR | 3 | 1.20e-01 | 1.00e+00 | 1.00e+00 | 1.00e+00 | 8.56e-01 |
| N1toR | 4 | 6.55e-01 | 6.25e-01 | 6.26e-01 | 1.00e+00 | 1.52e-01 |
| N2toW | 1 | **9.14e-04** | **7.27e-03** | **1.07e-02** | 4.64e-01 | **2.30e-05** |
| N2toW | 2 | **1.36e-08** | **1.07e-06** | **1.53e-09** | **2.85e-03** | **6.71e-11** |
| N2toW | 3 | **4.72e-04** | **6.56e-03** | **8.72e-04** | 2.25e-01 | **9.44e-06** |
| N2toW | 4 | **2.46e-07** | **9.00e-06** | **3.62e-06** | **1.20e-02** | **1.10e-09** |
| N2toN1 | 1 | **1.32e-02** | 4.26e-01 | **3.26e-02** | 3.04e-01 | **6.08e-04** |
| N2toN1 | 2 | **4.00e-06** | 9.80e-03 | **1.47e-05** | **1.07e-02** | **5.01e-08** |
| N2toN1 | 3 | **1.65e-07** | **1.97e-04** | **3.29e-06** | **9.60e-03** | **7.21e-10** |
| N2toN1 | 4 | **7.00e-09** | **1.95e-05** | **3.24e-07** | **3.62e-04** | **8.33e-12** |
| N2toN2 | 1 | **4.51e-04** | 1.40e-01 | **1.02e-03** | 7.32e-02 | **1.48e-05** |
| N2toN2 | 2 | 1.70e-01 | 7.72e-01 | 4.90e-01 | 4.09e-01 | **1.42e-02** |
| N2toN2 | 3 | **1.32e-02** | 3.87e-01 | 6.81e-02 | 4.82e-01 | **5.02e-04** |
| N2toN2 | 4 | **6.78e-15** | **3.91e-10** | **1.68e-13** | **6.23e-06** | **1.72e-18** |
| N2toN3 | 1 | 8.05e-01 | 1.00e+00 | 1.00e+00 | 1.00e+00 | 5.34e-01 |
| N2toN3 | 2 | 5.00e-01 | 9.59e-01 | 1.00e+00 | 1.00e+00 | 4.04e-01 |
| N2toN3 | 3 | 1.17e-01 | 1.00e+00 | 3.57e-01 | 1.00e+00 | 6.26e-01 |
| N2toN3 | 4 | **1.02e-03** | **1.37e-02** | **3.28e-04** | 1.05e-01 | **2.61e-05** |
| N2toR | 1 | **8.82e-03** | **2.52e-02** | 1.00e+00 | 1.00e+00 | 3.34e-01 |
| N2toR | 2 | **1.17e-05** | **3.70e-06** | 1.08e-01 | 1.00e+00 | **6.87e-05** |
| N2toR | 3 | **2.55e-03** | **2.05e-04** | 1.60e-01 | 1.24e-01 | **4.46e-04** |
| N2toR | 4 | 4.69e-01 | 1.00e+00 | 3.20e-01 | 1.00e+00 | 9.38e-02 |
| N3toW | 1 | 3.17e-01 | 1.00e+00 | 1.00e+00 | 6.10e-01 | 6.72e-01 |
| N3toW | 2 | 4.33e-02 | 1.00e+00 | 1.00e+00 | **4.38e-02** | 4.82e-01 |
| N3toW | 3 | 6.08e-01 | 1.00e+00 | 1.00e+00 | 1.00e+00 | 4.10e-01 |
| N3toW | 4 | 8.49e-01 | 1.00e+00 | 1.00e+00 | 1.00e+00 | 4.39e-01 |
| N3toN1 | 1 | 7.97e-01 | 1.00e+00 | 1.00e+00 | 1.00e+00 | 5.23e-01 |
| N3toN1 | 2 | 6.42e-01 | 1.00e+00 | 1.00e+00 | 1.00e+00 | 4.70e-01 |
| N3toN1 | 3 | 8.72e-01 | 1.00e+00 | 1.00e+00 | 1.00e+00 | 7.40e-01 |
| N3toN1 | 4 | 5.00e-01 | 1.00e+00 | 1.00e+00 | 1.00e+00 | 3.06e-01 |
| N3toN2 | 1 | 2.94e-01 | 1.00e+00 | 6.70e-01 | 7.17e-01 | 3.58e-02 |
| N3toN2 | 2 | 1.17e-01 | 1.00e+00 | 1.00e+00 | 7.01e-02 | 1.34e-01 |
| N3toN2 | 3 | 6.69e-01 | 1.00e+00 | 1.00e+00 | 1.00e+00 | 5.69e-01 |
| N3toN2 | 4 | 7.83e-01 | 1.00e+00 | 1.00e+00 | 1.00e+00 | 9.89e-01 |
| N3toN3 | 1 | 2.30e-01 | 7.11e-01 | 1.00e+00 | 5.78e-01 | **2.65e-02** |
| N3toN3 | 2 | 1.32e-02 | 3.24e-01 | **5.67e-03** | 7.18e-01 | **1.11e-03** |
| N3toN3 | 3 | 8.38e-01 | 1.00e+00 | 1.00e+00 | 1.00e+00 | 4.64e-01 |
| N3toN3 | 4 | 6.57e-02 | **3.35e-02** | 1.89e-01 | 5.47e-01 | **4.23e-03** |
| N3toR | 1 | 8.57e-01 | 1.00e+00 | 1.00e+00 | 1.00e+00 | 4.89e-01 |
| N3toR | 2 | 4.37e-01 | 2.32e-01 | 1.00e+00 | 1.00e+00 | 1.64e-01 |
| N3toR | 3 | 5.03e-01 | 1.00e+00 | 1.00e+00 | 6.08e-01 | 3.58e-01 |
| N3toR | 4 | 1.76e-01 | 2.39e-01 | 1.00e+00 | 1.00e+00 | 2.93e-01 |
| RtoW | 1 | 1.46e-01 | 2.67e-01 | 1.44e-01 | 4.35e-01 | **1.03e-02** |
| RtoW | 2 | 8.17e-02 | 1.00e+00 | 2.49e-01 | 3.04e-01 | **8.38e-03** |
| RtoW | 3 | **3.05e-04** | **1.31e-02** | **1.59e-03** | 1.63e-01 | **4.88e-06** |
| RtoW | 4 | **2.57e-05** | **3.19e-03** | **2.77e-05** | 2.49e-01 | **6.95e-07** |
| RtoN1 | 1 | **3.05e-03** | **2.93e-03** | **1.19e-02** | 7.02e-02 | **6.85e-05** |
| RtoN1 | 2 | **4.94e-03** | **9.29e-03** | **2.56e-03** | 1.00e+00 | **3.39e-04** |
| RtoN1 | 3 | **4.08e-03** | **6.71e-03** | **1.11e-03** | 1.37e-01 | **9.63e-05** |
| RtoN1 | 4 | **3.53e-06** | **5.40e-05** | **5.74e-07** | **2.27e-03** | **1.89e-08** |
| RtoN2 | 1 | **1.15e-02** | **2.79e-02** | 1.00e+00 | **6.99e-02** | **5.51e-03** |
| RtoN2 | 2 | 8.72e-01 | 1.00e+00 | 1.00e+00 | 1.00e+00 | 8.24e-01 |
| RtoN2 | 3 | 1.09e-01 | 5.60e-02 | 1.00e+00 | 1.00e+00 | 1.66e-01 |
| RtoN2 | 4 | 8.57e-01 | 1.00e+00 | 1.00e+00 | 1.00e+00 | 8.02e-01 |
| RtoN3 | 1 | 6.23e-01 | 1.00e+00 | 1.00e+00 | 1.00e+00 | 4.88e-01 |
| RtoN3 | 2 | NaN | NaN | NaN | NaN | NaN |
| RtoN3 | 3 | 6.55e-01 | 9.42e-01 | 8.58e-01 | 1.00e+00 | 1.50e-01 |
| RtoN3 | 4 | 7.41e-01 | 1.00e+00 | 1.00e+00 | 1.00e+00 | 3.31e-01 |
| RtoR | 1 | **1.87e-04** | 1.00e+00 | 8.47e-01 | 4.46e-05 | **2.14e-02** |
| RtoR | 2 | **1.40e-02** | **3.77e-02** | 1.77e-01 | 1.00e+00 | **1.03e-02** |
| RtoR | 3 | 1.46e-01 | 3.66e-01 | 5.95e-01 | 1.00e+00 | **1.06e-02** |
| RtoR | 4 | **3.52e-02** | 7.00e-01 | 3.98e-01 | 8.88e-01 | 1.98e-01 |

IH, idiopathic hypersomnia; N1-3, sleep stage N1-3; NT1, narcolepsy type 1; NT2, narcolepsy type 2; prop, proportion; R, REM sleep; SSI, stage shift index; TST, total sleep time; W, wake. Bold denotes statistical significance at the level of 0.05.

**Suppl Table 5: ANOVA analysis results for quarter-night qEEG features**

| Feature | Interval | anova_pVal | NT1_vs_NT2 | NT1_vs_IH | NT1_vs_Control | NT1_vs_ALL |
| --- | --- | --- | --- | --- | --- | --- |
| mean_delta_W | 1 | **2.14e-03** | 2.18e-01 | **3.25e-02** | 5.54e-01 | **2.91e-05** |
| mean_delta_W | 2 | **9.79e-09** | **5.76e-06** | **1.23e-05** | 1.50e-01 | **1.78e-11** |
| mean_delta_W | 3 | **6.95e-05** | **1.23e-02** | **4.77e-03** | 1.00e+00 | **4.02e-06** |
| mean_delta_W | 4 | **2.63e-02** | 5.91e-01 | 1.00e+00 | 1.00e+00 | **4.09e-02** |
| mean_theta_W | 1 | **2.81e-02** | 1.27e-01 | 1.49e-01 | 2.14e-02 | **1.42e-03** |
| mean_theta_W | 2 | **9.05e-03** | **3.21e-03** | 1.00e+00 | 3.43e-01 | **1.64e-03** |
| mean_theta_W | 3 | 4.46e-01 | 1.00e+00 | 1.00e+00 | 6.70e-01 | 2.16e-01 |
| mean_theta_W | 4 | 3.12e-01 | 3.12e-01 | 1.00e+00 | 1.00e+00 | 7.18e-02 |
| mean_alpha_W | 1 | **1.01e-02** | 1.44e-01 | 2.30e-01 | 1.00e+00 | **4.61e-04** |
| mean_alpha_W | 2 | **1.43e-06** | 8.52e-05 | 3.71e-04 | 4.52e-01 | **1.09e-08** |
| mean_alpha_W | 3 | **8.46e-05** | **2.60e-02** | **2.74e-02** | 1.00e+00 | **1.80e-04** |
| mean_alpha_W | 4 | **3.92e-02** | 9.28e-01 | 1.00e+00 | 8.07e-01 | 1.69e-01 |
| mean_sigma_W | 1 | 8.29e-01 | 1.00e+00 | 1.00e+00 | 1.00e+00 | 9.24e-01 |
| mean_sigma_W | 2 | 2.19e-01 | 1.00e+00 | 1.00e+00 | 1.00e+00 | 3.49e-01 |
| mean_sigma_W | 3 | 2.72e-01 | 1.00e+00 | 1.00e+00 | 1.80e-01 | 3.94e-01 |
| mean_sigma_W | 4 | **1.18e-02** | 7.61e-02 | **6.99e-03** | **3.19e-03** | **8.82e-04** |
| mean_beta_W | 1 | 1.68e-01 | 1.00e+00 | 1.00e+00 | 1.00e+00 | **3.75e-02** |
| mean_beta_W | 2 | **1.16e-04** | 1.49e-01 | 3.34e-03 | 1.00e+00 | **4.69e-06** |
| mean_beta_W | 3 | 1.06e-01 | 9.38e-01 | 6.30e-01 | 1.00e+00 | **9.74e-03** |
| mean_beta_W | 4 | 4.65e-01 | 1.00e+00 | 1.00e+00 | 1.00e+00 | 5.39e-01 |
| mean_gamma_W | 1 | **6.78e-05** | 6.11e-01 | **8.93e-05** | 3.59e-01 | **6.61e-06** |
| mean_gamma_W | 2 | **3.40e-06** | 7.34e-02 | **5.90e-06** | 1.46e-01 | **1.24e-07** |
| mean_gamma_W | 3 | **6.13e-06** | 1.33e-02 | **2.32e-06** | 9.31e-02 | **1.23e-07** |
| mean_gamma_W | 4 | **1.46e-03** | 8.57e-01 | **8.26e-04** | 1.74e-01 | **1.20e-04** |
| mean_delta_N1 | 1 | 7.98e-01 | 1.00e+00 | 1.00e+00 | 1.00e+00 | 4.15e-01 |
| mean_delta_N1 | 2 | **3.92e-02** | 2.78e-01 | 5.04e-01 | 1.00e+00 | **2.99e-03** |
| mean_delta_N1 | 3 | **2.14e-03** | 1.65e-01 | 5.27e-02 | 1.00e+00 | **9.23e-04** |
| mean_delta_N1 | 4 | 6.73e-02 | 6.99e-01 | 1.00e+00 | 1.00e+00 | 8.04e-02 |
| mean_theta_N1 | 1 | 2.22e-01 | 1.77e-01 | 1.00e+00 | 7.42e-01 | 1.25e-01 |
| mean_theta_N1 | 2 | **3.80e-02** | 8.78e-02 | 1.00e+00 | 1.07e-01 | **3.27e-03** |
| mean_theta_N1 | 3 | **2.96e-02** | 9.11e-02 | 7.81e-02 | 9.58e-02 | **7.87e-04** |
| mean_theta_N1 | 4 | **3.62e-02** | **3.34e-02** | **4.03e-02** | 2.73e-01 | **9.80e-04** |
| mean_alpha_N1 | 1 | 7.04e-01 | 1.00e+00 | 7.55e-01 | 1.00e+00 | 2.53e-01 |
| mean_alpha_N1 | 2 | **3.92e-02** | 2.87e-01 | 1.00e+00 | 1.00e+00 | 7.29e-02 |
| mean_alpha_N1 | 3 | **3.92e-02** | 7.14e-01 | 1.00e+00 | 1.00e+00 | 5.14e-02 |
| mean_alpha_N1 | 4 | 7.07e-02 | 1.00e+00 | 1.00e+00 | 4.48e-01 | 4.56e-01 |
| mean_sigma_N1 | 1 | **2.58e-02** | 1.00e+00 | 9.88e-02 | 3.31e-03 | **2.84e-02** |
| mean_sigma_N1 | 2 | 4.83e-01 | 1.00e+00 | 1.00e+00 | 8.00e-01 | 8.83e-01 |
| mean_sigma_N1 | 3 | 1.21e-01 | 1.00e+00 | 1.00e+00 | 8.71e-02 | 5.36e-01 |
| mean_sigma_N1 | 4 | **6.42e-03** | 7.17e-01 | 6.60e-01 | 3.01e-04 | **4.84e-02** |
| mean_beta_N1 | 1 | 1.92e-01 | 5.75e-02 | 2.35e-01 | 2.90e-01 | **2.73e-02** |
| mean_beta_N1 | 2 | 6.69e-01 | 1.00e+00 | 1.00e+00 | 1.00e+00 | 3.52e-01 |
| mean_beta_N1 | 3 | 1.09e-01 | 1.00e+00 | 1.00e+00 | 1.00e+00 | 2.51e-01 |
| mean_beta_N1 | 4 | 7.34e-01 | 1.00e+00 | 6.95e-01 | 1.00e+00 | 5.88e-01 |
| mean_gamma_N1 | 1 | **1.52e-02** | 1.00e+00 | **1.98e-02** | 1.00e+00 | **4.69e-02** |
| mean_gamma_N1 | 2 | 1.48e-01 | 1.00e+00 | 2.70e-01 | 1.00e+00 | 1.40e-01 |
| mean_gamma_N1 | 3 | 1.12e-01 | 1.00e+00 | 1.55e-01 | 1.00e+00 | 1.06e-01 |
| mean_gamma_N1 | 4 | 1.26e-01 | 1.00e+00 | 2.00e-01 | 2.58e-01 | **2.53e-02** |
| mean_delta_N2 | 1 | 4.53e-01 | 1.00e+00 | 1.00e+00 | 1.00e+00 | 2.46e-01 |
| mean_delta_N2 | 2 | **2.37e-04** | **9.86e-03** | **3.02e-03** | 5.07e-01 | **2.56e-06** |
| mean_delta_N2 | 3 | **2.14e-03** | 5.97e-02 | 1.33e-01 | 1.00e+00 | **1.40e-04** |
| mean_delta_N2 | 4 | 9.92e-02 | 7.92e-01 | 1.00e+00 | 1.00e+00 | **2.73e-02** |
| mean_theta_N2 | 1 | 7.81e-01 | 1.00e+00 | 1.00e+00 | 1.00e+00 | 5.98e-01 |
| mean_theta_N2 | 2 | 5.51e-02 | 1.36e-01 | 8.53e-01 | 1.00e+00 | **3.43e-03** |
| mean_theta_N2 | 3 | 5.10e-02 | 1.91e-01 | 8.03e-01 | 1.00e+00 | **9.88e-03** |
| mean_theta_N2 | 4 | 1.05e-01 | 2.92e-01 | 9.10e-01 | 1.00e+00 | **1.44e-02** |
| mean_alpha_N2 | 1 | 1.48e-01 | 1.00e+00 | 1.00e+00 | 5.33e-01 | 5.02e-01 |
| mean_alpha_N2 | 2 | **2.31e-02** | 4.03e-01 | 2.96e-01 | 1.00e+00 | **3.35e-03** |
| mean_alpha_N2 | 3 | **4.81e-02** | 1.00e+00 | 1.00e+00 | 1.00e+00 | **3.07e-02** |
| mean_alpha_N2 | 4 | 2.07e-01 | 1.00e+00 | 1.00e+00 | 1.00e+00 | 2.48e-01 |
| mean_sigma_N2 | 1 | 8.38e-01 | 1.00e+00 | 1.00e+00 | 1.00e+00 | 9.92e-01 |
| mean_sigma_N2 | 2 | 9.47e-01 | 1.00e+00 | 1.00e+00 | 1.00e+00 | 7.03e-01 |
| mean_sigma_N2 | 3 | 3.46e-01 | 1.00e+00 | 9.05e-01 | 1.00e+00 | 3.76e-01 |
| mean_sigma_N2 | 4 | **2.70e-02** | 5.94e-01 | **9.96e-03** | 2.65e-01 | **1.72e-03** |
| mean_beta_N2 | 1 | 9.21e-01 | 1.00e+00 | 1.00e+00 | 1.00e+00 | 8.44e-01 |
| mean_beta_N2 | 2 | **2.10e-03** | 1.74e-01 | **1.05e-03** | 3.82e-01 | **6.07e-05** |
| mean_beta_N2 | 3 | **2.14e-03** | 4.49e-01 | **4.64e-03** | 6.64e-01 | **8.91e-05** |
| mean_beta_N2 | 4 | 1.92e-01 | 1.00e+00 | 1.00e+00 | 1.00e+00 | **2.00e-02** |
| mean_gamma_N2 | 1 | **3.69e-04** | 1.00e+00 | **3.71e-04** | 6.05e-02 | **5.15e-05** |
| mean_gamma_N2 | 2 | **2.14e-03** | 2.32e-01 | **7.99e-04** | 1.09e-01 | **8.42e-05** |
| mean_gamma_N2 | 3 | **1.57e-03** | 3.63e-01 | **6.79e-04** | 5.98e-02 | **6.72e-05** |
| mean_gamma_N2 | 4 | **4.03e-03** | 2.38e-01 | **2.63e-03** | 6.58e-02 | **1.18e-04** |
| mean_delta_N3 | 1 | **3.92e-02** | 1.00e+00 | 1.00e+00 | 1.18e-01 | 5.99e-01 |
| mean_delta_N3 | 2 | **1.35e-04** | 1.30e-02 | **2.01e-03** | 1.00e+00 | **3.21e-05** |
| mean_delta_N3 | 3 | 9.54e-01 | 1.00e+00 | 1.00e+00 | 1.00e+00 | 5.40e-01 |
| mean_delta_N3 | 4 | 1.78e-01 | 1.00e+00 | 2.37e-01 | 1.65e-01 | 6.08e-02 |
| mean_theta_N3 | 1 | 1.84e-01 | 1.00e+00 | 1.00e+00 | 8.37e-01 | 4.87e-01 |
| mean_theta_N3 | 2 | **3.46e-03** | 6.29e-02 | **3.10e-02** | 1.00e+00 | **4.18e-04** |
| mean_theta_N3 | 3 | 9.21e-01 | 1.00e+00 | 1.00e+00 | 1.00e+00 | 6.07e-01 |
| mean_theta_N3 | 4 | 9.85e-02 | 4.67e-01 | 1.50e-01 | 5.70e-02 | **1.41e-02** |
| mean_alpha_N3 | 1 | 1.32e-01 | 1.00e+00 | 1.00e+00 | 1.45e-01 | 9.66e-01 |
| mean_alpha_N3 | 2 | **1.15e-02** | 5.34e-01 | 2.76e-01 | 1.00e+00 | **1.09e-02** |
| mean_alpha_N3 | 3 | 9.62e-01 | 1.00e+00 | 1.00e+00 | 1.00e+00 | 6.09e-01 |
| mean_alpha_N3 | 4 | 1.21e-01 | 1.00e+00 | 7.72e-02 | 9.65e-02 | 4.13e-02 |
| mean_sigma_N3 | 1 | 5.23e-01 | 1.00e+00 | 1.00e+00 | 1.00e+00 | 7.90e-01 |
| mean_sigma_N3 | 2 | 5.42e-02 | 2.83e-01 | 1.27e-01 | 1.00e+00 | **1.62e-02** |
| mean_sigma_N3 | 3 | 2.22e-01 | 1.00e+00 | 1.23e-01 | 1.00e+00 | 1.55e-01 |
| mean_sigma_N3 | 4 | 1.78e-01 | 1.00e+00 | 8.12e-02 | 1.00e+00 | 6.70e-02 |
| mean_beta_N3 | 1 | 1.32e-01 | 1.00e+00 | 1.00e+00 | 2.93e-01 | 9.77e-01 |
| mean_beta_N3 | 2 | 8.89e-02 | 3.08e-01 | 7.03e-02 | 1.00e+00 | **1.14e-02** |
| mean_beta_N3 | 3 | 9.78e-01 | 1.00e+00 | 1.00e+00 | 1.00e+00 | 9.14e-01 |
| mean_beta_N3 | 4 | 8.29e-01 | 1.00e+00 | 1.00e+00 | 1.00e+00 | 7.41e-01 |
| mean_gamma_N3 | 1 | 1.26e-01 | 1.00e+00 | 3.45e-01 | 1.00e+00 | 1.41e-01 |
| mean_gamma_N3 | 2 | 1.32e-01 | 4.07e-01 | 1.10e-01 | 1.00e+00 | **1.15e-02** |
| mean_gamma_N3 | 3 | 3.98e-01 | 1.00e+00 | 1.00e+00 | 1.00e+00 | 7.07e-02 |
| mean_gamma_N3 | 4 | 6.49e-01 | 1.00e+00 | 1.00e+00 | 1.00e+00 | 1.65e-01 |
| mean_delta_REM | 1 | **2.63e-02** | **6.09e-02** | 4.44e-01 | 5.83e-01 | **8.50e-04** |
| mean_delta_REM | 2 | 1.58e-01 | 4.61e-01 | 1.00e+00 | 1.00e+00 | 6.01e-02 |
| mean_delta_REM | 3 | 1.52e-01 | 5.36e-01 | 1.00e+00 | 1.00e+00 | **5.00e-02** |
| mean_delta_REM | 4 | 1.26e-01 | 5.98e-01 | 1.00e+00 | 1.00e+00 | **4.32e-02** |
| mean_theta_REM | 1 | 4.90e-01 | 1.00e+00 | 1.00e+00 | 1.00e+00 | 8.42e-01 |
| mean_theta_REM | 2 | 1.26e-01 | 1.00e+00 | 4.73e-01 | 3.98e-01 | 6.39e-02 |
| mean_theta_REM | 3 | 1.78e-01 | 1.00e+00 | 7.94e-01 | 8.74e-01 | 1.24e-01 |
| mean_theta_REM | 4 | 1.07e-01 | 1.00e+00 | 2.15e-01 | 1.00e+00 | 1.23e-01 |
| mean_alpha_REM | 1 | 1.96e-01 | 5.69e-01 | 1.00e+00 | 1.00e+00 | **3.06e-02** |
| mean_alpha_REM | 2 | 9.36e-02 | 1.44e-01 | 1.00e+00 | 1.00e+00 | **3.88e-02** |
| mean_alpha_REM | 3 | 1.02e-01 | 3.04e-01 | 1.00e+00 | 1.00e+00 | **4.53e-02** |
| mean_alpha_REM | 4 | 5.10e-02 | 1.75e-01 | 1.00e+00 | 1.00e+00 | **1.15e-02** |
| mean_sigma_REM | 1 | 9.21e-01 | 1.00e+00 | 1.00e+00 | 1.00e+00 | 7.31e-01 |
| mean_sigma_REM | 2 | 3.94e-01 | 1.00e+00 | 1.00e+00 | 1.00e+00 | 4.04e-01 |
| mean_sigma_REM | 3 | 2.75e-01 | 1.00e+00 | 1.00e+00 | 1.00e+00 | 3.00e-01 |
| mean_sigma_REM | 4 | 2.17e-01 | 1.00e+00 | 1.00e+00 | 3.91e-01 | 7.72e-01 |
| mean_beta_REM | 1 | **2.29e-02** | 4.52e-01 | 8.73e-02 | 5.80e-01 | **4.89e-04** |
| mean_beta_REM | 2 | **3.58e-02** | 1.00e+00 | 2.44e-01 | 3.33e-01 | **1.24e-03** |
| mean_beta_REM | 3 | **6.27e-02** | 1.00e+00 | 5.11e-01 | 2.57e-01 | **4.49e-03** |
| mean_beta_REM | 4 | **7.48e-02** | 1.00e+00 | 3.79e-01 | 1.00e+00 | **1.27e-02** |
| mean_gamma_REM | 1 | **1.83e-07** | 7.04e-03 | **8.66e-08** | **9.43e-04** | **1.19e-09** |
| mean_gamma_REM | 2 | **2.25e-06** | 1.60e-01 | **1.95e-05** | **3.11e-04** | **9.32e-08** |
| mean_gamma_REM | 3 | **4.09e-07** | 4.25e-01 | **5.97e-06** | **1.16e-04** | **5.65e-08** |
| mean_gamma_REM | 4 | **3.40e-06** | 5.77e-01 | **3.36e-06** | 7.99e-02 | **1.28e-06** |

IH, idiopathic hypersomnia; N1-3, sleep stage N1-3; NT1, narcolepsy type 1; NT2, narcolepsy type 2; prop, proportion; REM, rapid eye movement; SSI, stage shift index; TST, total sleep time; W, wake.

Bold denotes statistical significance at the level of 0.05.

**Suppl Table 6: ANOVA analysis results for quarter-night hypnodensity features**

| Feature | Interval | anova_pVal | NT1_vs_NT2 | NT1_vs_IH | NT1_vs_Control | NT1_vs_ALL |
| --- | --- | --- | --- | --- | --- | --- |
| W | 1 | **1.28e-05** | **3.21e-04** | **2.08e-03** | **3.42e-03** | **1.33e-07** |
| W | 2 | **9.91e-12** | **9.99e-09** | **6.60e-07** | **1.60e-07** | **1.36e-14** |
| W | 3 | **1.53e-08** | **2.10e-05** | **2.02e-05** | **5.24e-05** | **4.30e-11** |
| W | 4 | **9.47e-09** | **1.37e-06** | **3.90e-05** | **1.28e-04** | **2.81e-11** |
| N1 | 1 | **4.15e-02** | 4.16e-01 | 1.00e+00 | 8.80e-02 | **5.13e-03** |
| N1 | 2 | **2.88e-07** | **2.50e-03** | **4.87e-05** | **4.99e-05** | **3.41e-09** |
| N1 | 3 | **2.88e-07** | **7.95e-04** | **1.54e-03** | **2.78e-05** | **4.34e-09** |
| N1 | 4 | **1.99e-07** | **9.40e-05** | **1.12e-04** | **5.05e-05** | **1.28e-09** |
| N2 | 1 | **5.29e-04** | **3.63e-02** | **7.79e-03** | **1.23e-04** | **1.35e-04** |
| N2 | 2 | **3.99e-08** | **4.38e-04** | **3.57e-05** | **1.34e-07** | **3.05e-09** |
| N2 | 3 | **2.49e-08** | **3.57e-03** | **7.39e-07** | **4.07e-05** | **4.57e-10** |
| N2 | 4 | **6.07e-18** | **3.04e-10** | **9.77e-14** | **3.73e-11** | **3.00e-21** |
| N3 | 1 | **8.18e-03** | 5.28e-01 | 1.50e-01 | 4.19e-01 | **2.94e-04** |
| N3 | 2 | **9.98e-03** | 4.80e-01 | **4.04e-02** | 2.27e-01 | **5.08e-04** |
| N3 | 3 | 6.06e-01 | 1.00e+00 | 1.00e+00 | 1.00e+00 | 6.97e-01 |
| N3 | 4 | **3.91e-02** | **6.36e-03** | **3.50e-02** | 5.47e-01 | **2.86e-03** |
| R | 1 | **3.27e-03** | 1.00e+00 | 1.58e-01 | 5.42e-02 | 6.82e-02 |
| R | 2 | **1.18e-04** | **6.76e-05** | 6.40e-01 | 1.00e+00 | **2.45e-04** |
| R | 3 | **3.49e-06** | **6.17e-07** | **4.59e-02** | 9.03e-02 | **2.18e-06** |
| R | 4 | **3.55e-02** | **2.76e-02** | 1.00e+00 | 1.00e+00 | 5.81e-02 |
| WN1 | 1 | **4.01e-04** | **1.07e-02** | **2.56e-02** | **1.18e-02** | **7.42e-06** |
| WN1 | 2 | **1.23e-10** | **1.41e-06** | **1.00e-06** | **1.83e-07** | **3.62e-13** |
| WN1 | 3 | **1.90e-09** | **1.41e-05** | **9.39e-06** | **5.63e-07** | **9.73e-12** |
| WN1 | 4 | **1.23e-10** | **2.60e-07** | **4.21e-07** | **6.43e-07** | **1.90e-13** |
| WN2 | 1 | 9.23e-02 | 1.00e+00 | 1.00e+00 | 1.38e-01 | 3.72e-01 |
| WN2 | 2 | **3.38e-02** | 1.00e+00 | 1.00e+00 | **4.96e-02** | 2.88e-01 |
| WN2 | 3 | 5.95e-02 | 9.23e-01 | 1.00e+00 | 8.90e-02 | 1.76e-01 |
| WN2 | 4 | **4.17e-02** | 1.00e+00 | 1.00e+00 | 5.12e-02 | 1.84e-01 |
| WN3 | 1 | 5.56e-02 | 5.04e-01 | 1.00e+00 | 1.41e-01 | 4.18e-01 |
| WN3 | 2 | **3.68e-02** | 1.31e-01 | 1.00e+00 | 7.81e-02 | 1.37e-01 |
| WN3 | 3 | 1.01e-01 | 1.85e-01 | 1.00e+00 | 1.37e-01 | 6.96e-02 |
| WN3 | 4 | 9.15e-02 | 1.49e-01 | 1.00e+00 | 1.08e-01 | **4.05e-02** |
| WR | 1 | **1.30e-05** | 4.45e-01 | **1.29e-04** | **6.05e-04** | **5.58e-06** |
| WR | 2 | **8.18e-03** | 1.00e+00 | **3.95e-02** | **2.08e-02** | **1.54e-03** |
| WR | 3 | **2.61e-03** | 1.00e+00 | 5.32e-02 | **8.35e-03** | **5.51e-03** |
| WR | 4 | **3.24e-07** | **8.04e-03** | **5.51e-05** | **1.38e-05** | **1.26e-08** |
| N1N2 | 1 | 7.29e-01 | 1.00e+00 | 1.00e+00 | 1.00e+00 | 7.02e-01 |
| N1N2 | 2 | **2.08e-03** | 5.43e-01 | 5.23e-02 | **8.06e-03** | **1.63e-04** |
| N1N2 | 3 | **1.68e-03** | 1.12e-01 | 2.38e-01 | **4.86e-03** | **1.14e-04** |
| N1N2 | 4 | 8.05e-02 | 9.35e-01 | 1.00e+00 | 1.86e-01 | **2.27e-02** |
| N1N3 | 1 | 2.31e-01 | 1.00e+00 | 1.00e+00 | 4.99e-01 | 7.35e-01 |
| N1N3 | 2 | 9.23e-02 | 1.00e+00 | 1.00e+00 | 2.40e-01 | 5.12e-01 |
| N1N3 | 3 | 1.50e-01 | 1.00e+00 | 1.00e+00 | 3.30e-01 | 6.75e-01 |
| N1N3 | 4 | 9.38e-02 | 2.98e-01 | 1.00e+00 | 8.97e-02 | **3.59e-02** |
| N1R | 1 | **2.18e-02** | 1.00e+00 | 1.03e-01 | 6.95e-02 | **1.29e-02** |
| N1R | 2 | 8.22e-02 | 1.37e-01 | 1.00e+00 | 4.57e-01 | **4.05e-02** |
| N1R | 3 | 1.99e-01 | 1.17e-01 | 2.48e-01 | 5.27e-01 | **2.96e-02** |
| N1R | 4 | 6.64e-01 | 1.00e+00 | 1.00e+00 | 1.00e+00 | 4.42e-01 |
| N2N3 | 1 | **7.41e-03** | **3.12e-02** | 2.55e-01 | **1.17e-02** | **8.32e-04** |
| N2N3 | 2 | **1.02e-03** | 1.30e-01 | **2.87e-03** | **8.20e-03** | **5.07e-05** |
| N2N3 | 3 | 3.89e-01 | 1.00e+00 | 1.00e+00 | 1.00e+00 | 9.40e-02 |
| N2N3 | 4 | 6.64e-01 | 1.00e+00 | 1.00e+00 | 1.00e+00 | 5.28e-01 |
| N2R | 1 | **1.30e-03** | **1.08e-03** | 1.00e+00 | 1.00e+00 | **1.36e-02** |
| N2R | 2 | **1.48e-05** | **2.18e-05** | 7.91e-01 | 1.68e-01 | **3.79e-05** |
| N2R | 3 | **1.79e-04** | **1.26e-04** | 1.00e+00 | 1.65e-01 | **3.52e-04** |
| N2R | 4 | **1.72e-02** | **1.54e-02** | 1.00e+00 | 4.94e-01 | **7.56e-03** |
| N3R | 1 | 1.01e-01 | 1.93e-01 | 1.00e+00 | 1.00e+00 | 2.54e-01 |
| N3R | 2 | **4.17e-02** | 7.92e-02 | 1.00e+00 | 1.00e+00 | 7.23e-02 |
| N3R | 3 | 5.05e-02 | 1.81e-01 | 1.00e+00 | 1.00e+00 | 1.92e-01 |
| N3R | 4 | 8.87e-01 | 1.00e+00 | 1.00e+00 | 1.00e+00 | 9.29e-01 |
| entropy | 1 | 8.15e-02 | 1.00e+00 | 1.00e+00 | 5.05e-02 | 1.42e-01 |
| entropy | 2 | **4.17e-02** | 1.00e+00 | 1.00e+00 | **1.89e-02** | 5.17e-02 |
| entropy | 3 | **4.49e-03** | 4.79e-01 | 4.04e-01 | **1.85e-03** | **1.82e-03** |
| entropy | 4 | **2.31e-06** | **8.62e-04** | **3.50e-03** | **1.50e-05** | **1.11e-07** |

IH, idiopathic hypersomnia; N1-3, sleep stage N1-3; NT1, narcolepsy type 1; NT2, narcolepsy type 2; prop, proportion; SSI, stage shift index; R, REM sleep; TST, total sleep time; W, wake.

Bold denotes statistical significance at the level of 0.05.

**Suppl Table 7: Mixed model analysis results, showing features with significant quarter-night by diagnosis interactions**

|  |  |  | P-values for fixed effects terms | | |
| --- | --- | --- | --- | --- | --- |
| Feature | Category | Transform | Age | Diagnosis | Diagnosis by quarter night |
| propN1 | Proportion | SQ | **1.39E-02** | **3.97E-19** | **2.32E-07** |
| propN2 | Proportion | NONE | n.s. | **9.93E-17** | **1.55E-05** |
| propN3 | Proportion | SQ | **1.98E-02** | n.s. | **4.57E-07** |
| propR | Proportion | NONE | n.s. | **2.20E-05** | **2.24E-05** |
| delta_W | qEEG | NONE | **7.35E-08** | **8.60E-04** | **1.10E-04** |
| alpha_W | qEEG | LG | **1.54E-05** | **2.80E-02** | **2.93E-03** |
| sigma_W | qEEG | LG | **1.01E-03** | n.s. | **5.19E-03** |
| beta_W | qEEG | LG | **6.37E-08** | **1.09E-02** | **3.44E-02** |
| delta_N1 | qEEG | NONE | **7.42E-05** | n.s. | **1.28E-03** |
| alpha_N1 | qEEG | LG | **2.94E-03** | n.s. | **5.46E-03** |
| beta_N1 | qEEG | LG | **1.43E-03** | **3.30E-02** | **2.36E-02** |
| delta_N2 | qEEG | NONE | **2.69E-05** | n.s. | **6.75E-05** |
| theta_N2 | qEEG | NONE | **3.13E-02** | n.s. | **1.47E-03** |
| alpha_N2 | qEEG | LG | **1.00E-04** | n.s. | **2.45E-03** |
| sigma_N2 | qEEG | LG | n.s. | n.s. | **2.54E-06** |
| beta_N2 | qEEG | LG | **1.14E-05** | n.s. | **4.69E-09** |
| gamma_N2 | qEEG | LG | **2.60E-03** | **1.91E-08** | **1.55E-05** |
| delta_N3 | qEEG | NONE | **4.32E-07** | n.s. | **1.18E-08** |
| theta_N3 | qEEG | NONE | **3.08E-06** | n.s. | **5.44E-08** |
| alpha_N3 | qEEG | LG | **3.08E-06** | n.s. | **5.89E-09** |
| sigma_N3 | qEEG | LG | **3.87E-02** | n.s. | **5.44E-08** |
| beta_N3 | qEEG | LG | **4.32E-07** | n.s. | **5.78E-08** |
| gamma_N3 | qEEG | LG | **8.31E-06** | **1.33E-04** | **9.11E-06** |
| gamma_REM | qEEG | LG | **2.24E-04** | **2.15E-06** | **3.10E-03** |
| WN1 | Mixed Probability | SQ | **1.54E-04** | **2.45E-07** | **3.10E-03** |
| WN2 | Mixed Probability | LG | **5.28E-02** | n.s. | **4.92E-02** |
| WN3 | Mixed Probability | LG | **1.04E-02** | n.s. | **5.95E-04** |
| WR | Mixed Probability | SQ | n.s. | **4.78E-09** | **4.05E-03** |
| N1N2 | Mixed Probability | SQ | **1.54E-05** | n.s. | **6.05E-04** |
| N1N3 | Mixed Probability | LG | n.s. | n.s. | **3.20E-02** |
| N1R | Mixed Probability | SQ | n.s. | **3.87E-02** | **4.96E-06** |
| N2N3 | Mixed Probability | SQ | **1.84E-02** | **2.35E-02** | **1.28E-03** |
| WtoW | Transitions | NONE | **2.57E-05** | **9.54E-15** | **4.35E-02** |
| WtoN2 | Transitions | SQ | **4.33E-04** | **1.91E-08** | **6.36E-03** |
| WtoR | Transitions | SQ | n.s. | **1.56E-02** | **1.94E-02** |
| N1toN1 | Transitions | NONE | n.s. | **4.78E-09** | **1.29E-03** |
| N2toN2 | Transitions | NONE | **1.11E-02** | **4.34E-07** | **4.86E-02** |
| N2toN3 | Transitions | SQ | n.s. | n.s. | **2.32E-02** |
| N2toR | Transitions | SQ | n.s. | 2.95E-08 | **5.76E-04** |
| N3toW | Transitions | LG | n.s. | n.s. | **1.44E-02** |
| N3toN3 | Transitions | NONE | n.s. | n.s. | **3.10E-03** |
| RtoR | Transitions | NONE | n.s. | 1.43E-07 | 7.24E-08 |
|  |  |  |  |  |  |

R, REM sleep; REM, rapid eye movement; SSI, stage shift index; TST, total sleep time; W, wake.

Bold denotes statistical significance at the level of 0.05.

**Suppl Table 8.** Comparison of performance between training and testing

| Task | Mean AUC test set | STD AUC test set | Mean AUC training set | STD AUC training set |
| --- | --- | --- | --- | --- |
| NT1 vs Clinical controls | 0.96 | 0.03 | 0.98 | 0.02 |
| NT1 vs NT2 | 0.89 | 0.03 | 0.90 | 0.05 |
| NT1 vs IH | 0.94 | 0.03 | 0.95 | 0.02 |
| NT1 vs NT2+IH | 0.91 | 0.03 | 0.92 | 0.01 |
| NT1 vs All | 0.91 | 0.03 | 0.92 | 0.07 |

Mean values for AUC and corresponding standard deviations over the 200 runs for both training and testing using the all features set. Similar results were obtained using the other feature sets.

**References**

Altmann, A., Tolosi, L., Sander, O., & Lengauer, T. (2010). Permutation importance: a corrected feature importance measure. *Bioinformatics, 26*(10), 1340–1347. doi:10.1093/bioinformatics/btq134

Chawla, N. V., Bowyer, K. W., Hall, L. O., & Kegelmeyer, W. P. (2002). SMOTE: synthetic minority over-sampling technique. *J Artif Intell Res, 16*, 321–357. doi:10.1613/jair.953

Chen, Q., Cao, F., Xing, Y., & Liang, J. (2023). Evaluating Classification Model Against Bayes Error Rate. *IEEE Transactions on Pattern Analysis and Machine Intelligence, 45*(8), 9639-9653. doi:doi: 10.1109/TPAMI.2023.3240194.

Friedman, J. H. (2002). Stochastic gradient boosting. *Comput Stat Data Anal, 38*(4), 367–378. doi:10.1016/S0167-9473(01)00065-2

Guyon, I., Weston, J., Barnhill, S., & Vapnik, V. (2002). Gene selection for cancer classification using support vector machines. *Machine Learning, 46*(1), 389–422.

Hawkins, D. M., Basak, S. C., & Mills, D. (2003). Assessing model fit by cross-validation. *J Chem Inf Comput Sci, 43*(2), 579-586. doi:10.1021/ci025626i

Kuhn, M., & Johnson, K. (2013). *Applied Predictive Modelling.*  New York: Springer.
